# Adjuvant Temozolomide Chemotherapy With or Without Interferon Alfa Among Patients With Newly Diagnosed High-grade Gliomas: A Randomized Clinical Trial

**DOI:** 10.1001/jamanetworkopen.2022.53285

**Published:** 2023-01-27

**Authors:** Chengcheng Guo, Qunying Yang, Pengfei Xu, Meiling Deng, Taipeng Jiang, Linbo Cai, Jibin Li, Ke Sai, Shaoyan Xi, Hui Ouyang, Mingfa Liu, Xianming Li, Zihuang Li, Xiangrong Ni, Xi Cao, Cong Li, Shaoxiong Wu, Xiaojing Du, Jun Su, Xiaoying Xue, Yiming Wang, Gang Li, Zhiyong Qin, Hui Yang, Tao Zhou, Jinquan Liu, Xuefeng Hu, Jian Wang, Xiaobing Jiang, Fuhua Lin, Xiangheng Zhang, Chao Ke, Xiaofei Lv, Yanchun Lv, Wanming Hu, Jing Zeng, Zhenghe Chen, Sheng Zhong, Hairong Wang, Yinsheng Chen, Ji Zhang, Depei Li, Yonggao Mou, Zhongping Chen

**Affiliations:** 1 Department of Neurosurgery and Neuro-oncology, Sun Yat-Sen University Cancer Center, State Key Laboratory of Oncology in South China, Collaborative Innovation Center of Cancer Medicine, Guangzhou, China; 2 Department of Radiation, Sun Yat-Sen University Cancer Center, State Key Laboratory of Oncology in South China, Collaborative Innovation Center of Cancer Medicine, Guangzhou, China; 3 Department of Neurosurgery, The First Affiliated Hospital of Shenzhen University, Shenzhen Second People’s Hospital, Shenzhen, China; 4 Department of Neuro-oncology, Guangdong Sanjiu Brain Hospital, Guangzhou, China; 5 Department of Clinical Research, Sun Yat-Sen University Cancer Center, State Key Laboratory of Oncology in South China, Collaborative Innovation Center of Cancer Medicine, Guangzhou, China; 6 Department of Pathology, Sun Yat-Sen University Cancer Center, State Key Laboratory of Oncology in South China, Collaborative Innovation Center of Cancer Medicine, Guangzhou, China; 7 Department of Neurosurgery, Guangdong Sanjiu Brain Hospital, Guangzhou, China; 8 Department of Neurosurgery, Shantou Central Hospital, Shantou, China; 9 Department of Radiation Oncology, Shenzhen People’s Hospital, The Second Clinical Medical College, Jinan University, Shenzhen, Guangdong, China; 10 The First Affiliated Hospital, Southern University of Science and Technology, Shenzhen, Guangdong, China; 11 Department of Neurosurgery, The Second Affiliated Hospital of Guangzhou University of Chinese Medicine, Guangzhou, China; 12 Guangdong Province Hospital of Chinese Medical, Guangzhou, China; 13 Department of Neurosurgery, Tumor Hospital of Harbin Medical University, Harbin, China; 14 Department of Radiotherapy, The Second Hospital of Hebei Medical University, Shijiazhuang, China; 15 Department of Medical Oncology, The First Affiliated Hospital, Jinan University, Guangzhou, China; 16 Department of Neurosurgery, Tangdu Hospital, Fourth Military Medical University, Xi’an, China; 17 Department of Neurosurgery, Huashan Hospital, Shanghai Medical College, Fudan University, Shanghai, China; 18 Neurosurgical Institute of Fudan University and Shanghai Clinical Medical Center of Neurosurgery, Shanghai, China; 19 Shanghai Key Laboratory of Brain Function and Restoration and Neural Regeneration, Shanghai, China; 20 Department of Neurosurgery, Xinqiao Hospital, Third Military Medical University, Chongqing, China; 21 Department of Oncology, Guangdong Armed Police Corps Hospital, Guangzhou, China; 22 Department of Radiation Oncology, Affiliated Cancer Hospital and Institute of Guangzhou Medical University, Guangzhou, China; 23 Department of Radiation Oncology, First People’s Hospital of Fo Shan Affiliated with Sun Yat-Sen University, Foshan, China; 24 Department of Medical Imaging, Sun Yat-Sen University Cancer Center, State Key Laboratory of Oncology in South China, Collaborative Innovation Center of Cancer Medicine, Guangzhou, China

## Abstract

**Importance:**

High-grade gliomas (HGGs) constitute the most common and aggressive primary brain tumor, with 5-year survival rates of 30.9% for grade 3 gliomas and 6.6% for grade 4 gliomas. The add-on efficacy of interferon alfa is unclear for the treatment of HGG.

**Objectives:**

To compare the therapeutic efficacy and toxic effects of the combination of temozolomide and interferon alfa and temozolomide alone in patients with newly diagnosed HGG.

**Design, Setting, and Participants:**

This multicenter, randomized, phase 3 clinical trial enrolled 199 patients with newly diagnosed HGG from May 1, 2012, to March 30, 2016, at 15 Chinese medical centers. Follow-up was completed July 31, 2021, and data were analyzed from September 13 to November 24, 2021. Eligible patients were aged 18 to 75 years with newly diagnosed and histologically confirmed HGG and had received no prior chemotherapy, radiotherapy, or immunotherapy for their HGG.

**Interventions:**

All patients received standard radiotherapy concurrent with temozolomide. After a 4-week break, patients in the temozolomide with interferon alfa group received standard temozolomide combined with interferon alfa every 28 days. Patients in the temozolomide group received standard temozolomide.

**Main Outcomes and Measures:**

The primary end point was 2-year overall survival (OS). Secondary end points were 2-year progression-free survival (PFS) and treatment tolerability.

**Results:**

A total of 199 patients with HGG were enrolled, with a median follow-up time of 66.0 (95% CI, 59.1-72.9) months. Seventy-nine patients (39.7%) were women and 120 (60.3%) were men, with ages ranging from 18 to 75 years and a median age of 46.9 (95% CI, 45.3-48.7) years. The median OS of patients in the temozolomide plus interferon alfa group (26.7 [95% CI, 21.6-31.7] months) was significantly longer than that in the standard group (18.8 [95% CI, 16.9-20.7] months; hazard ratio [HR], 0.64 [95% CI, 0.47-0.88]; *P* = .005). Temozolomide plus interferon alfa also significantly improved median OS in patients with O6-methylguanine-DNA methyltransferase (MGMT) unmethylation (24.7 [95% CI, 20.5-28.8] months) compared with temozolomide (17.4 [95% CI, 14.1-20.7] months; HR, 0.57 [95% CI, 0.37-0.87]; *P* = .008). Seizure and influenzalike symptoms were more common in the temozolomide plus interferon alfa group, with 2 of 100 (2.0%) and 5 of 100 (5.0%) patients with grades 1 and 2 toxic effects, respectively (*P* = .02). Finally, results suggested that methylation level at the *IFNAR1/2* promoter was a marker of sensitivity to temozolomide plus interferon alfa.

**Conclusions and Relevance:**

Compared with the standard regimen, temozolomide plus interferon alfa treatment could prolong the survival time of patients with HGG, especially the MGMT promoter unmethylation variant, and the toxic effects remained tolerable.

**Trial Registration:**

ClinicalTrials.gov Identifier: NCT01765088

## Key Points

**Question:**

Does interferon alfa enhance the clinical benefits of temozolomide as the first-line treatment in patients with newly diagnosed high-grade glioma (HGG)?

**Findings:**

In this phase 3 randomized clinical trial study of 199 patients with HGG, compared with temozolomide alone, temozolomide plus interferon alfa significantly improved the overall survival of patients with HGG, especially those with O6-methylguanine-DNA methyltransferase (MGMT) unmethylation, which met the primary overall survival end point. The methylation level at the *IFNAR1/2* promoter was a marker of sensitivity to temozolomide plus interferon alfa.

**Meaning:**

Compared with the standard regimen, temozolomide plus interferon alfa treatment could prolong the survival time of patients with HGG with tolerable toxic effects, especially among patients with the MGMT promoter unmethylatation.

## Teaser

This randomized clinical trial evaluates the efficacy of the combination of temozolomide with interferon alfa in patients with newly diagnosed high-grade glioma.

## Introduction

High-grade gliomas (HGGs) are defined as World Health Organization (WHO) grade 3 or grade 4 gliomas and mainly include glioblastoma (GBM), gliosarcoma, anaplastic glioma, anaplastic oligodendroglioma, and anaplastic oligoastrocytoma.^1^ The current standard treatment consists of maximal surgical tumor resection followed by fractionated radiotherapy and 6 cycles of temozolomide-based chemotherapy.^2,3,4,5,6^ Despite aggressive treatment, the long-term survival of patients with HGG is still not promising, with 5-year overall survival (OS) of 30.9% for grade 3 gliomas and 6.6% for grade 4 gliomas. Moreover, patients with an unmethylated promoter for the gene encoding O6-methylguanine-DNA methyltransferase (MGMT) had a more aggressive prognosis and resistance to temozolomide,^7^ with a median progression-free survival (PFS) of 5.3 to 6.9 months in patients with GBM. Methylation of MGMT not only changes the biology of a tumor but also affects its vulnerability to temozolomide.

Interferon alfa has been associated with innate immune system antiviral response and is regarded as a naturally occurring glycoprotein with immunomodulatory, antiproliferative, and antiangiogenic effects. Also, interferon alfa could have some interaction with the blood-brain barrier and have the antitumor activity in malignant neoplasms. Although the retrospective studies^8,9,10,11,12^ showed the response rates of interferon alfa were as high as 40% in patients with glioma, dose management, treatment interval, and combination administration are still not confirmed. A previous study by Shen et al^13^ has revealed that interferon alfa markedly enhanced the efficacy of temozolomide in MGMT-positive glioma stemlike cells. Moreover, MGMT expression is markedly decreased with the combination of temozolomide and interferon alfa. A previous study^14^ of 30 patients with recurrent HGG who received the combination treatment of temozolomide and interferon alfa indicated that the combination therapy might have moderate activity in treating HGG. Therefore, we initiated a randomized, multicenter, phase 3 clinical trial to confirm the efficacy of the combination of temozolomide with interferon alfa in newly diagnosed HGG.

## Methods

### Study Design and Patient Selection

This randomized, multicenter, phase 3 clinical trial (the CSNO2012001 study) was initiated to compare the efficacy of combined temozolomide and interferon alfa with temozolomide alone in patients with newly diagnosed HGG. All patients provided written informed consent before participation in the study. The informed consent form and trial protocol (available in Supplement 1) were approved by the Chinese Society of Neuro-oncology and the ethics committees of the participating centers. This study followed the Consolidated Standards of Reporting Trials (CONSORT) reporting guideline.

Patients aged 18 to 75 years with newly diagnosed HGG (WHO grades 3 and 4 astrocytomas, including supratentorial GBM, gliosarcoma, anaplastic gliomas, anaplastic oligoastrocytomas, and anaplastic oligodendroglioma) were enrolled. Patients who had received no prior chemotherapy, radiotherapy, or immunotherapy for their brain tumor and had WHO Karnofsky performance status of at least 60% and normal organ function were included.

### Treatment Plan

Within 6 weeks after surgery, eligible patients were randomly assigned into the combined treatment group (temozolomide plus interferon alfa) or the standard treatment group (temozolomide alone). All patients received standard radiotherapy concurrent with temozolomide (Temodar; MDS China Holding Co, Ltd) at a dose of 75 mg/m^2^/d for 42 days with a standard fractionated radiotherapy (60 Gy). After a 4-week break, the patients in the combined treatment group received interferon alfa (3 million U on days 1, 3, and 5) plus temozolomide (150-200 mg/m^2^ on days 2-6) every 28 days for a maximum of 12 cycles. Patients in the standard treatment group received temozolomide (150-200 mg/m^2^ on days 1-5) every 28 days for a maximum of 12 cycles. The patients were followed up every 2 months (ie, after every 2 cycles of chemotherapy). Disease progression was evaluated based on the Response Assessment in Neuro-oncology criteria.^15,16^ Archival or fresh tumor biopsy samples were prospectively obtained from patients prior to treatment and confirmed the methylation status of the MGMT promoter^17^ (eMethods in Supplement 2).

### Clinical Outcome

The primary end point of the study was the 2-year OS. The time from the date of surgery until the time of death or the last follow-up visit was defined as OS. The 2-year PFS and treatment tolerability were used as secondary end points. Toxic effects were measured according to the National Cancer Institute Common Terminology Criteria for Adverse Events (version 3.0). Follow-up was conducted every 2 months during the treatment, and posttreatment follow-up was conducted every 3 months for the next 3 years and thereafter every 6 months until death or the end of the study. Preset subgroup analysis included WHO grade 3 or 4 and MGMT methylation status. During the treatment period, safety and disease assessments were performed regularly according to the schedule of activities for each arm. Treatment continued in both arms until progressive disease, death, unacceptable toxic effects, the start of a new anticancer therapy, withdrawal of consent, or the end of the study, whichever occurred first. Dose interruptions or reductions may have been required following potential drug toxicities.

### Exome Sequence Data Processing and Mutation Calling

To identify molecular features that were significantly enriched in either responsive or nonresponsive tumors, we collected tumor and blood samples from 20 patients in the temozolomide plus interferon alfa group, which was divided into the responder group and nonresponder group. Patients were classified as responders if the tumor was either stable or shrinking continually over at least 6 courses of treatment. The tumor tissues and matching blood samples analyzed in this study were obtained from the biospecimen bank of Sun Yat-Sen University Cancer Center. Detection of the whole exon sequencing, DNA methylation analysis, and RNA sequencing data analysis were performed^18,19,20,21,22,23,24,25,26^ (eMethods in Supplement 2).

### Statistical Analysis

The primary objective of this trial was to test whether temozolomide plus interferon alfa improved OS compared with temozolomide alone. Based on previous reports,^1,27^ we assumed that the 2-year OS was 35% for patients treated with temozolomide alone and 52% for patients treated with temozolomide plus interferon alfa, meaning an absolute improvement of 17% in 2-year OS with a target hazard ratio (HR) of 0.62. The expected length of accrual period and the expected maximum length of follow-up were both 42 months. After accounting for a 15% dropout rate, approximately 194 patients (97 per group) would be required to achieve 80% power at a 2-sided type I error of .05, with 142 events expected for the primary analysis of OS.

Data were analyzed from September 13 to November 24, 2021. All analyses were performed based on an intention-to-treat population. Permuted block with a flexible block size (4 or 6) was used to generate the randomization allocation sequence. Randomization was stratified by pathological findings (grade 3 or 4). The random allocation sequences were generated and maintained by an independent, unblinded statistician from a third-party vendor. The patient randomization and the dispensing of investigational drugs were implemented via the Interactive Web Response System (Octalsoft).

Survival outcomes were calculated using the Kaplan-Meier method. Survival differences were compared using a log-rank test. Adjustments of the significance threshold were performed for secondary end points and subgroup. Bivariable and multivariable analyses were conducted using the Cox proportional hazards regression model to investigate the effects of different survival factors. We used the χ^2^ test to determine the differences in the incidence of complications and peritreatment mortality. A 2-sided *P* value of less than .05 indicated a statistically significant finding for all analyses. Statistical analyses were performed using SPSS, version 22.0 (IBM Corp).

## Results

### Patient Characteristics

**Figure 1.**
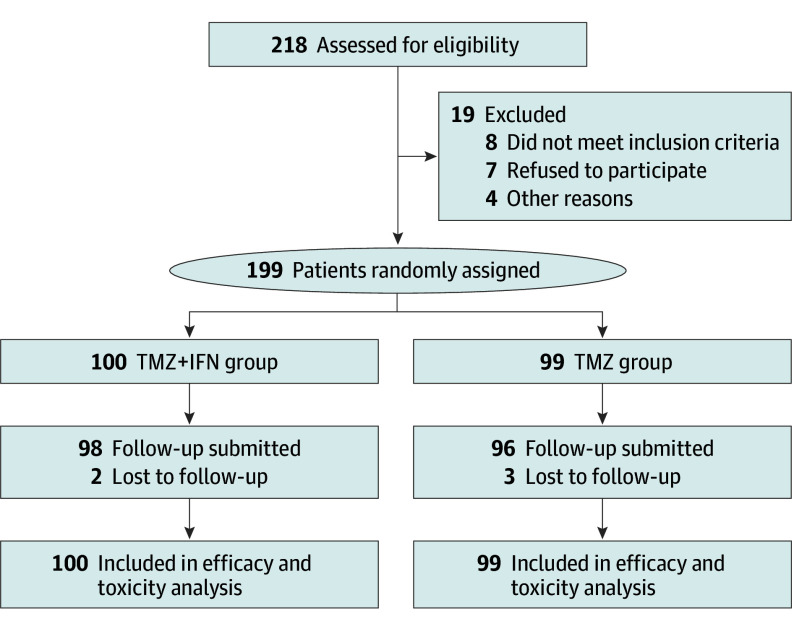
Study Flow Diagram Overview of screened and randomly assigned patients. TMZ + IFN indicates temozolomide plus interferon alfa.

From May 1, 2012, to March 30, 2016, a total of 199 patients from 15 Chinese centers (Figure 1 and eTable 1 in Supplement 2) were eligible and enrolled in our study (120 men [60.3%] and 79 women [39.7%]; median age, 46.9 [45.3-48.7] years). The baseline characteristics were balanced between the 2 groups (Table). Patients were randomized into the temozolomide plus interferon alfa group (n = 100) or temozolomide alone group (n = 99).

**Table.** Clinical Characteristics of the Patients in the Temozolomide Chemotherapy Plus Interferon Alfa Cohort and Temozolomide Alone Cohort^a^

Characteristic	Grade 3 glioma		*P* value	Grade 4 glioma		*P* value
	Temozolomide plus interferon alfa (n = 47)	Temozolomide alone (n = 44)		Temozolomide plus interferon alfa (n = 53)	Temozolomide alone (n = 55)	
Age, median (range), y	46.0 (19-74)	46.5 (25-71)	.50	46.0 (19-75)	47.0 (25-70)	.19
Sex						
Men	31 (66.0)	26 (59.1)	.50	30 (56.6)	33 (60.0)	.72
Women	16 (34.0)	18 (40.9)		23 (43.4)	22 (40.0)	
KPS, median (95% CI), %	78.4 (69.5-87.3)	76.4 (76.5-85.4)	.93	75.5 (65.8-87.0)	74.1 (64.4-82.6)	.56
Resection						
Total	32 (68.1)	28 (63.6)	.66	35 (66.0)	29 (52.7)	.16
Partial	15 (31.9)	16 (36.4)		18 (34.0)	26 (47.3)	
No. of cycles, median (95% CI)	6.0 (5.4-7.3)	6.0 (5.4-7.4)	.09	6.0 (5.5-7.2)	6.0 (5.6-7.5)	.60
Pathological finding						
Anaplastic astrocytoma	27 (57.4)	28 (63.6)	.55	NA	NA	NA
Anaplastic oligodendroglioma	16 (34.0)	11 (25.0)	.35	NA	NA	NA
Anaplastic oligoastrocytoma	4 (8.5)	5 (11.4)	.60	NA	NA	NA
MGMT status						
Methylation	22 (46.8)	18 (40.9)	.57	26 (49.1)	26 (47.3)	.85
Unmethylation	25 (53.2)	26 (59.1)		27 (50.9)	29 (52.7)	
Follow-up, median (95% CI), mo	67.7 (55.0-80.5)	53.3 (4.2-66.5)	.10	66.0 (37.9-94.1)	61.5 (58.9-65.2)	.95
PFS, median (95% CI), mo	24.3 (21.7-27.0)	14.1 (9.8-18.5)	.04	12.0 (9.8-14.2)	12.8 (12.2-13.4)	.58
OS, median (95% CI), mo	39.6 (35.0-44.1)	29.4 (24.9-33.9)	.04	20.5 (16.5-24.6)	17.7 (15.4-20.0)	.04

Abbreviations: KPS, Karnofsky performance status; MGMT, methylguanine-DNA methyltransferase; NA, not applicable; OS, overall survival; PFS, progression-free survival.

^a^ Unless otherwise indicated, data are expressed as No. (%) of patients.

### Efficacy

After a follow-up for a median duration of 66.0 (95% CI, 59.1-72.9) months, completed on July 31, 2021, 181 patients (91.0%) showed progression, and 165 (82.9%) died due to tumor progression. The median number of cycles in the temozolomide plus interferon alfa group was 6.0 (95% CI, 5.2-6.8); in the temozolomide alone group, 6.0 (95% CI, 5.4-6.6). A total of 150 patients (75.4%) received long-term treatment (≥6 cycles), and the ratio of those receiving long-term treatment to those who did not showed no difference between the temozolomide plus interferon alfa and temozolomide groups (72 of 100 [72.0%] vs 78 of 99 [78.8%]; *P* = .18).

**Figure 2.**
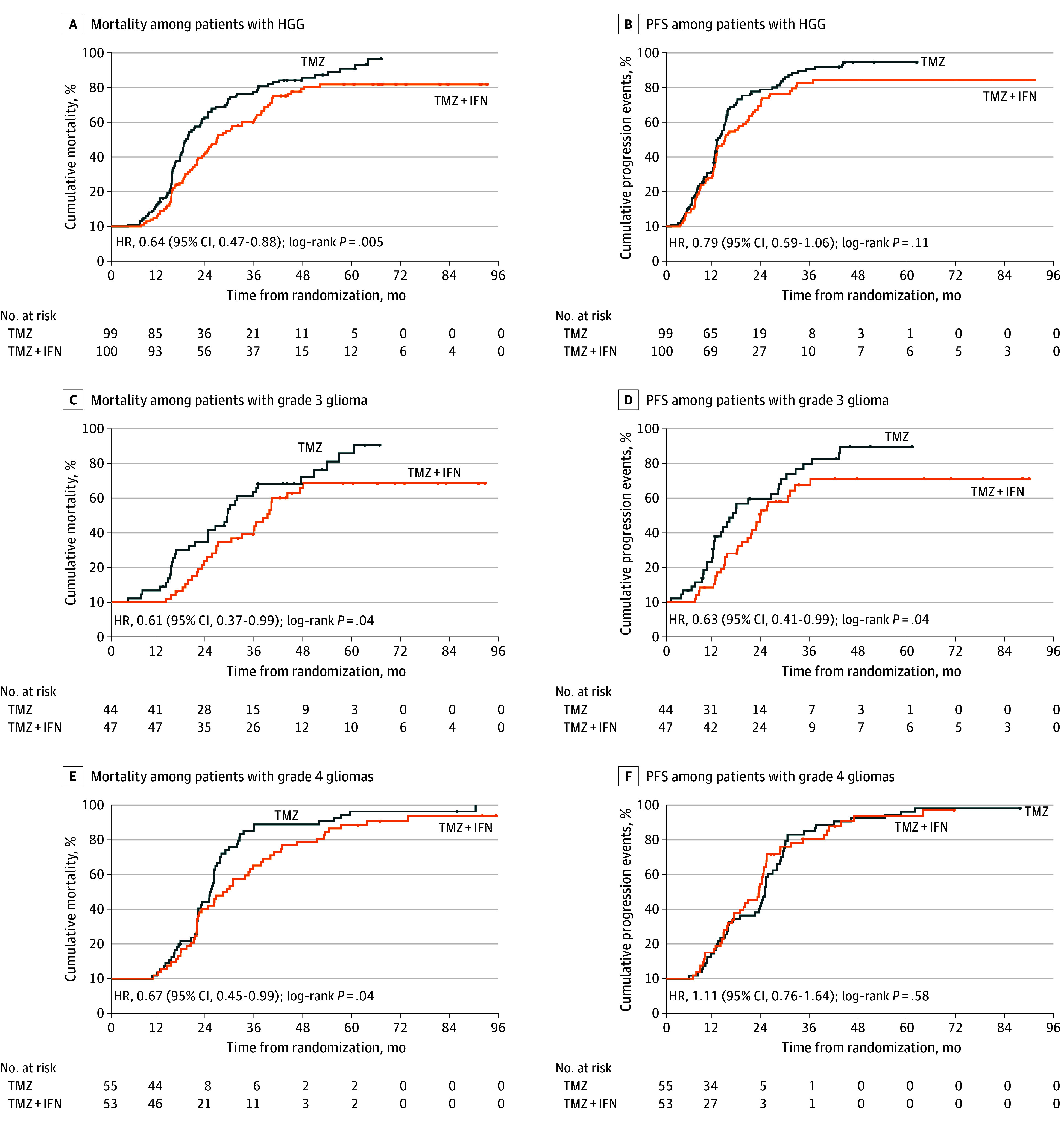
Survival Among Patients With High-grade Glioma (HGG) Treated With Temozolomide Plus Interferon Alfa (TMZ + IFN) Compared With Temozolomide (TMZ) Alone HR indicates hazard ratio; PFS, progression-free survival.

As the primary end point, the median OS of the temozolomide plus interferon alfa group (26.7 [95% CI, 21.6-31.7] months) was significantly prolonged compared with the temozolomide group (18.8 [95% CI, 16.9-20.7] months; HR, 0.64 [95% CI, 0.47-0.88]; *P* = .005) (Figure 2A). The median 2-year OS rates were 57.4% (95% CI, 47.6%-67.2%) in the temozolomide plus interferon alfa group vs 37.3% (95% CI, 27.7%-46.9%) in the temozolomide group. The median 5-year OS rates were 18.1% (95% CI, 10.1%-26.1%) vs 9.1% (95% CI, 2.4%-15.8%), respectively. When we analyzed patients with grade 3 and grade 4 gliomas separately, the median OS was also longer in the temozolomide plus interferon alfa group (WHO grade 3, 39.6 [95% CI, 35.0-44.1] months; WHO grade 4, 20.5 [95% CI, 16.5-24.6] months) compared with the temozolomide alone group for WHO grade 3 gliomas (29.4 [95% CI, 24.9-33.9] months; HR, 0.61 [95% CI, 0.37-0.99]; *P* = .04) (Figure 2C) and WHO grade 4 glioma (17.7 [95% CI, 15.4-20.0] months; HR, 0.67 [95% CI, 0.45-0.99]; *P* = .04) (Figure 2E).

As the secondary end point, the median PFS showed no significant difference between the temozolomide plus interferon alfa group (14.8 [95% CI, 12.3-17.4] months) and temozolomide group (12.9 [95% CI, 11.8-14.0] months; HR, 0.79 [95% CI, 0.59-1.06]; *P* = .11) (Figure 2B). The median 2-year PFS rates were 27.9% (95% CI, 19.1%-36.7%) in the temozolomide plus interferon alfa group vs 18.5% (95% CI, 10.9%-26.1%) in the temozolomide group. The median 5-year PFS rates were 9.6% (95% CI, 3.5%-15.7%) in the temozolomide plus interferon alfa group vs 4.8% (95% CI, 0.5%-9.1%) in the temozolomide group. However, in grade 3 gliomas, the median PFS was longer in the temozolomide plus interferon alfa group (24.3 [95% CI, 21.7-27.0] months) than in the temozolomide group (14.1 [95% CI, 10.1-18.2] months; HR, 0.63 [95% CI, 0.41-0.99]; *P* = .04) (Figure 2D). In grade 4 gliomas, the difference in median PFS between the temozolomide plus interferon alfa group (12.0 [95% CI, 9.8-14.2] months) and temozolomide group (12.8 [95% CI, 12.2-13.4] months) showed no significant difference (HR, 1.11 [95% CI, 0.76-1.64]; *P* = .58) (Figure 2F).

**Figure 3.**
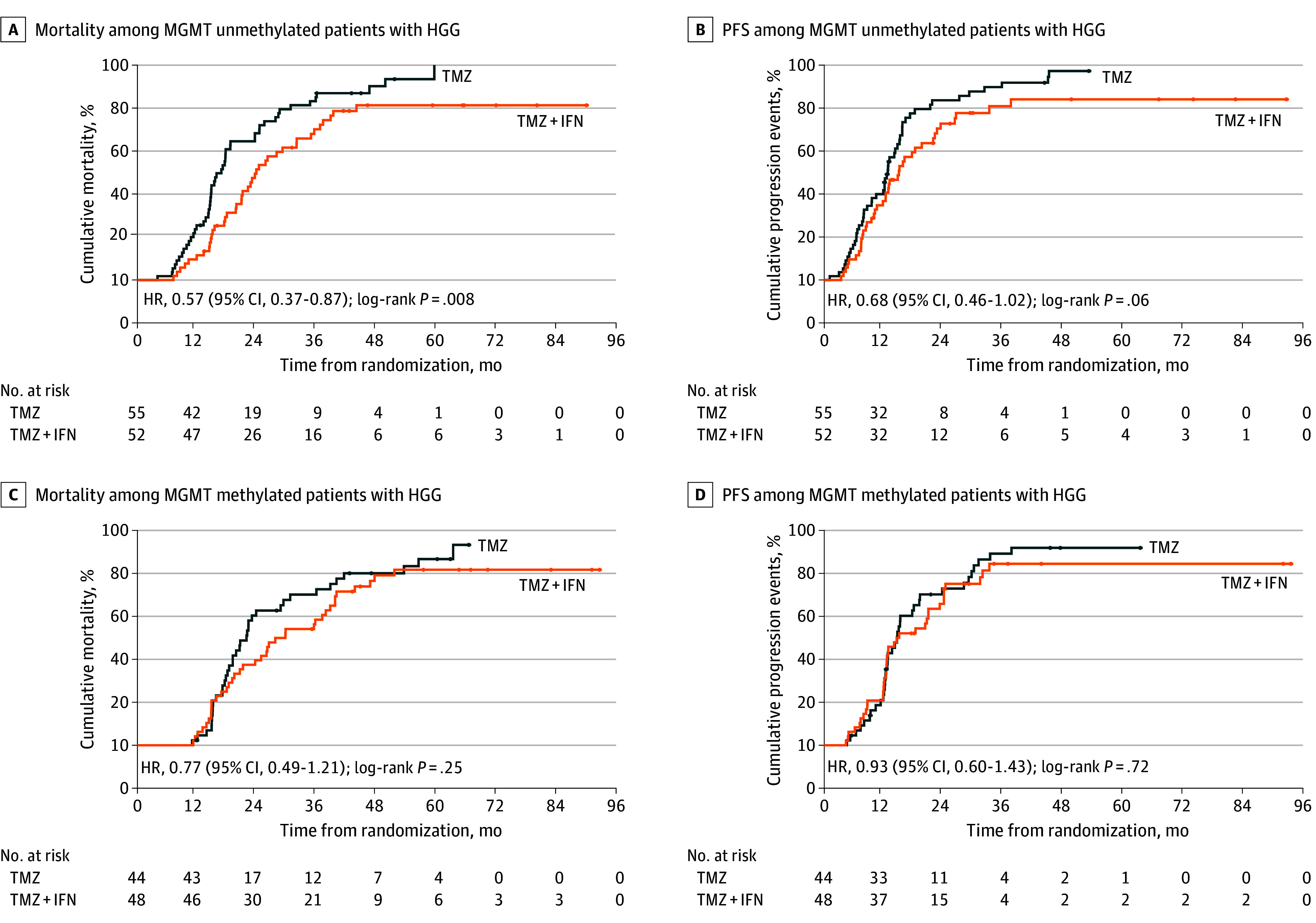
Survival Among Patients With High-grade Glioma (HGG) by O6-Methylguanine-DNA Methyltransferase (MGMT) Status Treatment groups included temozolomide plus interferon alfa (TMZ + IFN) and temozolomide alone (TMZ). PFS indicates progression-free survival; HR, hazard ratio.

In MGMT-related subgroup analysis, temozolomide plus interferon alfa treatment showed significant improvement in the median OS of patients with MGMT unmethylation (24.7 [95% CI, 20.5-28.8] months in the temozolomide plus interferon alfa group vs 17.4 [95% CI, 14.1-20.7] months in the temozolomide group; HR, 0.57 [95% CI, 0.37-0.87]; *P* = .008) (Figure 3A), while there was no statistical difference in median OS in the MGMT methylation subgroup between the 2 treatment groups (28.3 [95% CI, 17.4-39.2] months in the temozolomide plus interferon alfa group vs 22.4 [95% CI, 19.2-25.6] months in the temozolomide group; HR, 0.77 [95% CI, 0.49-1.21]; *P* = .25) (Figure 3C). There was no difference in median PFS between patients with MGMT unmethylation (14.8 [95% CI, 11.6-18.0] months in the temozolomide plus interferon alfa group vs 12.6 [95% CI, 11.8-13.5] months in the temozolomide group; HR, 0.68 [95% CI, 0.46-1.02]; *P* = .06) (Figure 3B) and in patients with MGMT methylation (14.7 [95% CI, 8.7-20.7] months in the temozolomide plus interferon alfa group vs 14.4 [95% CI, 11.9-16.9] months in the temozolomide group; HR, 0.93 [95% CI, 0.60-1.43]; *P* = .72) (Figure 3D).

The bivariable and multivariable analyses are shown in eTable 3 in Supplement 2. The treatment group, WHO Karnofsky performance status, the extent of tumor resection, MGMT status, and the pathological grade of the tumor remained as risk factors for OS independently of other factors.

### Toxic Effects

Toxic effects were evaluated in all 199 patients, and no grade 4 toxic effects were found. Most were modest in general. However, seizure and influenzalike symptoms such as fever, chill, or headaches were more common in the temozolomide plus interferon alfa group, with 2 of 100 patients (2.0%) with grade 1 toxic effects and 5 of 100 patients with (5.0%) with grade 2 toxic effects (*P* = .02). One patient (1.0%) developed grade 3 influenzalike symptoms after the first cycle of temozolomide plus interferon alfa, which required withdrawal from the study group and receipt of temozolomide alone (eTable 2 in Supplement 2). Adverse events leading to the discontinuation of temozolomide plus interferon alfa or temozolomide placebo occurred in 1 of 100 (1.0%) and 0 patients, respectively.

### Association of Methylation Level at the *IFNAR1/2* Promoter With Temozolomide Plus Interferon Alfa Responders

All 20 tumor samples underwent whole-exon sequencing analysis, 15 for DNA methylation analysis, and 13 for transcriptome analysis (eFigure, A in Supplement 2). Although the mutational profiles were similar between responder and nonresponder groups (eFigure, B in Supplement 2), we found that the methylation level at the *IFNAR1/2* promoter (probes cg00937568 and cg23202109) in the nonresponder group was significantly higher than in the responder group (median for probe cg00937568, 0.140 [IQR, 0.132-0.161] vs 0.099 [IQR, 0.093-0.105], respectively; median for probe cg23202109, 0.127 [IQR, 0.107-0.147] vs 0.067 [IQR, 0.052-0.072], respectively; *P* < .001) (eFigure, C and D in Supplement 2). The proportion of samples with MGMT promoter methylation was similar between the 2 groups (2 of 6 in the responder group vs 4 of 9 in the nonresponder group; *P* = .78) (eFigure, C in Supplement 2). Consistent with these results, the responder group had the higher messenger RNA expression of *IFNAR1* (median in the responder group, 22.1 [IQR, 18.8-25.8]; median in the nonresponder group, 11.2 [IQR, 10.5-13.7]; *P* = .02) and *IFNAR2* (median in the responder group, 11.1 [IQR, 8.7-12.8]; median in the nonresponder group, 5.6 [IQR, 4.5-7.0]; *P* = .03) (eFigure, E and F in Supplement 2), suggesting that the methylation level at the *IFNAR1/2* promoter was potentially a marker of sensitivity to temozolomide plus interferon alfa. In addition, the results of gene set enrichment analysis also confirmed that several gene sets were associated with treatment response, including *TNFA/NFKB* signaling (adjusted *P* = .004), *IL6/JAK/STAT3* signaling (adjusted *P* = .004), apoptosis (adjusted *P* = .004), interferon gamma response (adjusted *P* = .004), *IL2/STAT5* signaling (adjusted *P* = .004), and interferon alfa response (adjusted *P* = .005). All of these gene sets contributed to interferon response according to previous studies (eFigure, G in Supplement 2).

## Discussion

In this randomized clinical trial, OS was significantly prolonged in the temozolomide plus interferon alfa group compared with the temozolomide group. Since the prognosis is different between patients with grade 4 and grade 3 gliomas,^28^ our survival analysis was separated. The OS and PFS for grade 3 glioma in the combination group were better, showing a trend of prolonged survival time. In patients with GBM, OS in the combined treatment group was significantly better, although PFS seems to be similar between the 2 treatment groups. Since there are no accurate diagnostic criteria for disease progression or recurrence, it is difficult to measure the PFS accurately and to distinguish between disease progression or recurrence and pseudoprogression. Nevertheless, interferon alfa as an immunotherapy might take longer to produce sustained tumor shrinkage and lead to unconventional response patterns not properly captured by the standard response assessments.^29,30,31,32^ As a result, standard PFS evaluation may not be the best way to capture antitumor activity of immunotherapy.^33^ Accurate OS can be measured because it was the length measured from the date of diagnosis to the date of death or the last follow-up. This might explain why the OS was significantly longer in the combination therapy group, but PFS showed no significant difference.

Promising immunotherapy was limited in glioma as a “cold tumor.” To our knowledge, this study investigates one of the combination therapies that may confirm the efficacy of immune-related treatment in gliomas. Interferon alfa can directly inhibit tumor cells’ proliferation, enhance the cytotoxic activity of macrophages and natural killer cells, and prevent the formation of blood vessels in tumors. Moreover, it can enhance the cytotoxic effect with S phase stagnation.^34,35,36^ Interferon alfa can sensitize the glioma stemlike cells by modulating MGMT expression through nuclear factor–κB inhibitory activity, enhancing the cytotoxic activity and reversing the resistance of temozolomide.^13^ Third, interferon alfa could modify the host’s immune response against tumor-inducing programmed cell death 1 ligand 1 upregulation, which could indirectly reactivate the antitumor immunity.^37,38^ Last but not least, interferon alfa could stimulate the production of type I interferon in endothelial cells of the blood-brain barrier. The combination of interferon alfa 1 and the heterodimeric receptor *IFNAR* produces a cellular response, which promotes heterodimers *STAT1/2* nuclear translocation and transcriptional activation of interferon-stimulated genes. The rapid expression of hundreds of interferon-stimulated genes is critical for controlling the biological function.^39^ Clinically, Groves et al^12^ determined the efficacy of a pegylated formulation of interferon alfa 2b and temozolomide in patients with recurrent GBM, which showed a median 6-month PFS of 31% to 38%, demonstrating some benefits over the standard use of temozolomide. A retrospective study from Japan^27^ confirmed interferon beta and temozolomide for patients with newly diagnosed primary GBM achieved a greater OS of 19.9 months when compared with 12.7 months for standard temozolomide treatment, particularly in patients with unmethylated MGMT promoter with prolonged OS of 17.2 months, which supported our study findings. However, the Japan Clinical Oncology Group Brain Tumor Study Group (JCOG-BTSG)^40^ demonstrated that the OS and PFS did not benefit in the temozolomide plus interferon beta group compared with temozolomide alone in patients with newly diagnosed GBM. Our study may have shown some differences in findings from the JCOG-BTSG study for several reasons. One potential reason is that the sensitized mechanism is different between the 2 subtypes of interferon. Second, compared with 1 dose of interferon in the JCOG-BTSG study, 3 doses of interferon in each cycle may increase the dose-dense treatment. Third, fewer cases of residual disease were found in our study than in the JCOG-BTSG study, which might hint that complete resection benefits combination treatment. Subgroup analyses in the JCOG-BTSG study also showed that interferon beta could possibly benefit patients with no residual tumor, supporting our hypothesis. In addition, our patients had less severe toxic effects than those in the JCOG-BTSG study, suggesting better tolerance of interferon, and maintenance of interferon use might benefit the treatment.

It was found that interferon alfa and beta have markedly enhanced chemosensitivity to temozolomide^13,41,42,43^ by downregulating MGMT expression.^42,44^ A mechanistic study^45^ showed that interferon alfa and beta suppressed nuclear factor–κB activity by inducing the p53 signaling pathway. Our clinical study results are consistent with those of the previous laboratory studies,^13,14^ suggesting that patients with unmethylated GBM benefit more from interferon combined with temozolomide chemotherapy. In addition, our results also showed that methylation level at the *IFNAR1/2* promoter was associated with responders to temozolomide plus interferon. *IFNAR1/2* was a virtually ubiquitous membrane receptor that binds endogenous type I interferon cytokines. The antiproliferative response has been reported to require high levels of *IFNAR* expression and occupancy.^46^ To our knowledge, we have the first report of the association between *IFNAR1/2* promoter and interferon responsiveness in the tumor treatment. Concordantly, such defects in interferon signaling may partially explain why only some patients benefit from interferon therapy.

The adverse effects could be evaluated in 199 patients, with no severe events observed. Influenzalike symptoms such as fatigue or myalgia and epilepsy were more common in the combination group, but both were controllable.

### Limitations

This study has some limitations. The CSNO2012001 study only included Chinese patients, which limited external validity toward other racial and ethnic groups. In addition, anaplastic oligodendroglioma as a subgroup of grade 3 gliomas with a relatively good prognosis may be a potential bias in our study. Furthermore, the molecular profiling of tumors was not performed, and molecular biology experimental validation should be performed in the future.

## Conclusions

In this randomized clinical trial, therapy consisting of temozolomide combined with interferon alfa prolonged the survival time of patients with newly diagnosed HGG, especially those with MGMT unmethylated tumors, compared with the standard temozolomide regimen, and the toxic effects remained tolerable. Thus, we suggest that patients with MGMT unmethylated HGG receive temozolomide plus interferon alfa combination treatment.

## Notes

Supplement 1.
Trial Protocol

Supplement 2. **eTable 1.** Participating Institution and Researchers **eTable 2.** Toxic Effects of the Patients in Temozolomide Plus Interferon Alfa Chemotherapy Cohort and Temozolomide Cohort **eTable 3.** Bivariable and Multivariable Analysis for OS and PFS **eMethods.** The Detection for the Methylation Status of the MGMT Promoter **eFigure.** Molecular Signatures Related to Response to Temozolomide With Interferon Alfa Therapy

Supplement 3.
Data Sharing Statement
