# Supplementary material for: Adjuvant Temozolomide Chemotherapy With or Without Interferon Alfa Among Patients With Newly Diagnosed High-grade Gliomas: A Randomized Clinical Trial
Source: JAMA Netw Open. 2023 Jan 27;6(1):e2253285. doi: 10.1001/jamanetworkopen.2022.53285 (PMC11839150; doi:10.1001/jamanetworkopen.2022.53285)
Supplement: Supplement 1. — Trial Protocol [file jamanetwopen-e2253285-s001.pdf]

Postoperative temozolomide standard regimen versus standard regimen combination for newly diagnosed high-grade gliomas
A multicenter randomized controlled clinical study of interferon alpha therapy

Chinese Anti-Cancer Association Neuro-Oncology Professional Committee Project: CSNO2012001

A Phase III Trial on Adjuvant Standard Temozolomide Chemotherapy with or without Interferon-alpha in Newly Diadnosed Malignant Gliomas.

Date of proposal writing: June 6, 2012

Research Background

World Health Organization (WHO) classification of central nervous system tumors, gliomas are classified into grades I to IV, of which grades III and IV are high-grade gliomas, also known as malignant gliomas. Grade III gliomas mainly include anaplastic a strocytoma (AA), anaplastic oligodendroglioma (AOG), anaplastic oligodendroglioma (anaplastic oligodendroglioma , AOG) oligoastrocytomas , AOA), grade IV gliomas mainly include glioblastoma multiforme (g lioblastoma m ultiforme , GBM) and gliomasarcomatosum (gliomasarcomatosum). High-grade glioma is the most common primary brain tumor in adults, accounting for 77.5% of all gliomas, with difficult treatment

and poor prognosis [1] . The median survival of newly diagnosed grade III and IV gliomas was 12 to 24 months and 8 to 15 months, respectively. In recent years, studies have shown that standard treatment based on surgery, supplemented by radiotherapy and chemotherapy after surgery can improve the survival of patients with malignant glioma.

Temozolomide (TMZ) is a new type of alkylating agent. After entering the body, it is widely distributed throughout the body without being metabolized by the liver. It can also pass through the blood-brain barrier and enter the cerebrospinal fluid to achieve effective drug concentration in the central nervous system. Its cerebrospinal fluid/plasma drug concentration ratio is close to 30% to 40%. In 1999, the U.S. Food and Drug Administration (FDA) approved it for recurrent anaplastic astrocytoma, and in 2005, the FDA approved it for newly diagnosed glioblastoma . In 2005, the European Organization for Research on Treatment of Cancer / National Cancer Institute of Canada (European Organization for Research on Treatment of Cancer / National Cancer Institute of Canada , EORTC / NCIC) jointly initiated a multi-center phase III randomized controlled clinical study,and its results showed that compared with postoperative radiotherapy alone, postoperative radiotherapy combined with TMZ concurrent chemotherapy and adjuvant chemotherapy can significantly prolong the survival time of newly diagnosed GBM patients, the median survival time increased from 12.1 months to 14.6 months, the 2-year survival rate increased from 10.4% to 26.5%, and the 5-year survival rate increased from 1.9% to 9.8%. Also, patients with the presence of O^{6} - methylguanine-DNA methyltransferase (O^{6} - methylguanine-DNA methyltransferase, MGMT) promoter methylation in tumor tissue obviously benefited more from the combination therapy. At present, the National Comprehensive Cancer Network (NCCN) oncology clinical practice guidelines, the "Chinese Central Nervous System Glioma Diagnosis and Treatment Consensus" formulated by the Oncology Professional Group of the Neurosurgery Branch of the Chinese Medical Association , 2010 China The "Outline of Diagnosis and Treatment of Common Tumors of the Central Nervous System" formulated by the Neuro-Oncology Professional Committee of the Anti-Cancer Association [1, 4], both recommend radiotherapy combined with TMZ concurrent chemotherapy (75mg/(m^{2}.d), d_{1-42}) and 6 cycles of adjuvant TMZ routine regimen for 5 days every 28 days(200 mg/(m^{2}.d), d_{1-5}) is used as the standard treatment after surgery for newly diagnosed GBM. For the postoperative adjuvant chemotherapy of grade III glioma, temozolomide or nitrosourea drugs can be selected, but oral administration of temozolomide has fewer adverse reactions and is more convenient to use. However, for patients who can continue to benefit from temozolomide chemotherapy and have tolerable toxicity, the guidelines recommend that an extended course of temozolomide, that is, longer than 6 courses of long-cycle chemotherapy, may be considered. For example, in the two clinical studies RTOG 0525 and RTOG 0825, for newly diagnosed glioblastoma patients, temozolomide adjuvant chemotherapy is recommended for up to 12 cycles. In RTOG0525, survival in both groups (conventional 5-day temozolomide regimen and dose-dense regimen) appeared to be higher than in the original EORTC/NCIC trial (median survival was 18.9 months and 16.9 months, respectively, higher than the EORTC/NCIC trial 14.6 months), and had no increase in toxic and side effects .

But despite this, the overall prognosis of malignant glioma is still not optimistic. With the current standard TMZ regimen including concurrent chemotherapy and adjuvant chemotherapy, the 5-year survival rate of newly diagnosed GBM patients is still less than 10%. Resistance to radio/chemotherapy mainly arises from DNA repair mechanisms within tumor cells. O^{6} -methylguanine -DNA methyltransferase (O^{6}-methylguanine-DNA methyltrans s ferase , MGMT) is a DNA repair protein encoded by the MGMT gene , which is transferred to its own cysteine residue, so that the alkylated

guanine on DNA is reduced, and itself becomes the inactive alkylated MGMT . It has been proved that MGMT is a relatively positive indicator associated with the resistance of malignant gliomas to nitrosourea and temozolomide. Hegi et al. detected the methylation status of the MGMT gene promoter in 206 patients in the EORTC/NCIC clinical study, and found that methylated patients could better benefit from radiotherapy combined with temozolomide chemotherapy [5] . Theoretically, methylation of the MGMT gene promoter can silence the MGMT gene and cause the MGMT protein to not be expressed. Some studies have shown that the expression of MGMT is significantly positively correlated with its enzymatic activity at the mRNA and protein levels. Immunohistochemical methods are often used to detect MGMT in clinical practice. Protein content to indirectly measure MGMT enzyme activity [6] . However, there was not complete agreement between MGMT gene promoter status and immunohistochemical protein expression. Further research found that in the mechanism of alkylating agent resistance in glioma, another key factor that does not depend on the methylation of MGMT gene promoter: nuclear factor kappaB (NF-κB) may be related to MGMT adjustment related. NF-κB is closely related to the occurrence, development and maintenance of cancer stem cells. Lavon et al reported [7] , NF-κB can induce the expression of MGMT protein, resulting in human glioma cell lines resistant to alkylating agents. The study found that there are two specific NF-κB binding sites in the MGMT promoter region, and both of these binding sites can specifically and directly bind to NF-κB. The expression of NF-κB subunit p65 in tumor cells can induce the expression of MGMT, the degree of activation of NF-κB is significantly positively correlated with the expression of MGMT, and the tumor cells with high degree of NF-κB activation are more susceptible to nitrosourea treatment. Not sensitive.

Interferon (interferon , IFN) is a class of important cytokines, which have pleiotropic biological functions such as anti-virus, inhibiting tumor growth, inhibiting angiogenesis, and regulating immune function. According to gene sequence, chromosomal location and receptor specificity can be divided into three types. Type I includes IFN -α , -β , -ω , -ε , -κ and other subtypes; type II is IFN -γ, also known as immune interferon; type III is a newly discovered cytokine IFN-λ. Although its complex mechanism has not been fully elucidated, IFN has been widely used in clinical practice such as treatment of viral infectious diseases such as hepatitis B and C, first-line treatment of malignant tumors such as malignant melanoma, hairy cell leukemia and non-Hodgkin's lymphoma, autoimmune diseases such as multiple sclerosis, etc. IFN can not only regulate the growth and division of somatic cells, but also affect the growth of cells by regulating apoptosis, and its biological effects are mainly mediated by the activation of the JAK/STAT signal transduction pathway. Oligonucleotide microarray studies have shown that more than 300 genes are induced by IFN treatment, some of which are critical for inducing apoptosis and controlling cell growth, especially IFN -mediated genes, which can directly affect the therapeutic effect of IFN [8] . Some studies have shown that in recurrent malignant glioma, the efficacy of IFN-α is related to the content of IFNα gene in tumor tissue, and the time to progress (TTP) of patients with low IFNα gene content is longer [9] . IFN -α is the most commonly used interferon in clinical tumor treatment, with an in vivo half-life of 4 to 16 h . IFN -α can directly inhibit the proliferation of tumor cells, enhance the killing activity of macrophages and NK cells on tumors, and can prevent the formation of blood vessels inside the tumor, stop the tumor from proliferating, and cause tumor cell necrosis [10] . In addition , IFN -α can also make more tumor cells stay in the S phase and cannot divide, thereby enhancing the cytotoxic effect of chemotherapy drugs, and has no cross toxicity with traditional chemotherapy drugs. An in vitro study by Sun Yat-Sen University Cancer Center showed that IFN -α or IFN - β (IFNα /β) combined with TMZ could significantly inhibit the

growth of glioma cells. In the growth inhibition experiment, IFN α/β treated MGMT-positive glioma stem cells to improve the chemosensitivity of TMZ, and the killing effect was significantly enhanced; RT-PCR and Western-blot detection results showed that MGMT positive after INFα/ β treatment The expression of NF- κB and MGMT in glioma stem cells was significantly reduced at the mRNA and protein levels [11]. In further in vivo experiments, the establishment of a subcutaneous tumor model in nude mice also proved that TMZ combined with IFN α/β had significantly higher efficacy than TMZ alone. At the same time , the results of immunohistochemistry and western blotting confirmed that the protein expressions of MGMT and NF-κB in the transplanted tumor tissues of nude mice were decreased after drug action, and the protein expression of the combination group was significantly lower than that of the TMZ alone group, suggesting that the synergistic effect of IFNα /β on TMZ is related to its inhibition of NF-κB protein expression in gliomas and then down-regulation of MGMT expression. The experiment found that IFNα /β combined with TMZ had better therapeutic effect on human glioma cells in nude mice subcutaneously transplanted than TMZ alone , which provided a new experimental basis for the clinical treatment of glioma [12].

In clinical research, many attempts have been made to use IFN -α alone or in combination with chemotherapy in lung cancer, melanoma, and gynecological tumors. In 1996 , Liao Meilin et al reported that low-dose IFN - α (1mIU/time, 2-3 times / week , course of treatment more than 6 months) postoperative immunotherapy compared with postoperative chemotherapy , can improve the 3 -year survival rate of patients with stage I non-small cell lung cancer. [13]. However,Yang Xuening et al demonstrated that the preliminary results of a randomized controlled clinical study of a randomized controlled clinical study of 44 patients with stage I and II non-small cell lung cancer who used with interferon-α after complete resection showed that IFN -αused alone (3mIU/time, intramuscular injection , once every 2 days, the entire course of treatment for a total of 90 days) did not significantly improve the survival rate . The authors analyzed that INF -α in this program requires frequent and long-term injections , with obvious local side effects and high costs [14].

In the clinical treatment of glioma, there have been some reports on the treatment of malignant glioma with IFNα /β alone or in combination with radiotherapy/chemotherapy since the 1990s. In a phase I/II clinical study conducted by Dillman et al. [15] in 1995, 17 newly diagnosed patients with malignant glioma received radiotherapy combined with IFNα - 2a (starting dose of 3 mIU/time, subcutaneous injection, 3 times a week, the dose was increased to 5 mIU/time after 2 weeks, up to 16 weeks) treatment. The median age of the patients was 64 years (range 24-77 years); it included 12 cases of GBM, 7 cases of advanced astrocytoma; 2 cases of complete resection, 9 cases of partial resection, and 8 cases of biopsy. The results were that two patients received radiotherapy combined with IFN - α therapy for less than 2 weeks due to rapid tumor progression, 1 patient discontinued IFN - α therapy due to rash, 1 patient discontinued treatment due to pneumonia, and 1 patient withdrew from the group due to poor compliance. . Fourteen patients completed 8 weeks of RT combined with IFN - α , but only 3 completed 16 weeks of IFN - α . The only grade IV adverse reaction was increased aspartate aminotransferase (3 cases), other adverse reactions included increased alkaline phosphatase (2 cases) and severe fatigue (4 cases). The median time to treatment failure was 2 months, and the median OS was 7.5 months. 4 cases survived for more than 1 year (the longest was 29.1 months, and 1 case was still alive after 20.7 months). In 1995, Buckner initiated a phase II clinical study [16] that 35 patients with astrocytoma, oligoastrocytoma and oligodendroglioma who had relapsed and progressed after previous radiotherapy (without chemotherapy) received BCNU combined with IFN -α treatment (IFN -α 12 mI U/m^2 , d1-3, BCNU 150 mg/m^2 on d3, repeated every 6 weeks). Its result illustrated that the

objective response rate was 29%, 37% of those patients had stable tumors for more than 6 months, and 25% of them had tumors less than 6 months . The median effective duration was 9.9 months, and the median overall survival time was 13.3 months. The main adverse reactions included : moderate myelosuppression, venous irritation symptoms, vomiting, flu-like symptoms and transiently reversible neurological worsening. In a phase II clinical study conducted by Brandes et al. [17] in 1997 , 21 patients with high-grade gliomas who had recurred after postoperative radiotherapy (without chemotherapy) received BCNU combined with IFN -α therapy (intravenous infusion on the first day of chemotherapy, IFN -α 6 mIU , followed by instillation of BCNU 150 mg/m ^{2}, followed by subcutaneous injection of IFN -α every other day , 3 mI U /time, 3 times a week, 42 days for a course of treatment). Results: Partial remission in 7 cases (33%) , stable in 6 cases (29%) , median TTP of 4.5 months, and median overall survival (OS) of 7 months. Median TTP and median OS were 9 months and 15 months, respectively, for patients who had undergone reoperation before chemotherapy, and 4 months and 5.5 months, respectively, for those without surgery, with tolerable toxicity . In a phase I clinical study of Rajkumar et al. [18] in 1998, radiotherapy combined with IFN -α and BCNU chemotherapy was used as the first-line treatment for high-grade glioma. Patients with grade III/IV glioma were enrolled after operation. At the beginning of radiotherapy, they received BCNU (200 mg/m ^{2} , d1) chemotherapy once, and BCNU (150 mg/m ^{2} , d1) chemotherapy was given once at the end of chemotherapy. At the same time, those patients received IFN -α therapy (12 mIU /m ^{2} , subcutaneous injection, 3 consecutive days per week, d1-3) at the beginning of radiotherapy , and the course of treatment was repeated every 7 weeks . The dose can be adjusted later according to the patient's systemic symptoms and myelosuppression caused by BCNU. Results: the toxicity and efficacy of 11 patients among 15 enrolled patients were evaluated. Major non-hematologic toxicities (which were also dose-limiting) included somnolence and flu-like symptoms. Due to the high toxicity, the IFN -α of the last 5 patients was adjusted to the 1-3 days of the 1st, 3rd and 5th weeks, and the course of treatment was repeated every 7 weeks. Adverse reactions such as somnolence, fever, chills, myalgia, alopecia, and anorexia were reported in all patients, and other adverse reactions included nausea/vomiting (91%) , depression or mood changes (64%), headache (55%), and elevated transaminases (36%), 4 cases (45%) of grade III/IV leukopenia , and 3 cases (27%) of grade III/IV thrombocytopenia . The median survival time was 44 months, the objective response rate was 56% , and the median duration of response was 33 months. In a phase III clinical study conducted by Buckner et al. [19] in 2001, 275 patients with newly diagnosed high-grade gliomas without tumor progression after postoperative radiotherapy were randomized to receive BCNU alone or BCNU combined with INF-α chemotherapy (BCNU 200 mg/m2). ^{2} ,d1,± INF-α 12 mI U/ m ^{2} , d1-3 , W1, 3, 5, repeat courses every 7 weeks, up to 6 courses). The results showed that the incidence of fever, chills, myalgia, and neurological symptoms was higher in the combined treatment group, and there was no significant difference in median TTP and median OS between the two groups.

Based on the above research reports from the 1990s to the early 20th century, IFN -α combined with BCNU is mostly used for treatment, and the dosage of IFN -α is large and the medication time is long. While BCNU is a traditional nitrosourea chemotherapy drug, its dose-limiting toxicity is severe myelosuppression and gastrointestinal reactions. Therefore, most patients have severe side effects and poor treatment tolerance, which also affects the survival of patients to a certain extent. Although nitrosoureas have been used in glioma for a long time, their role in adjuvant chemotherapy for newly diagnosed malignant gliomas has not been confirmed in prospective randomized controlled clinical studies. Temozolomide is a new type of alkylating agent. In 2005, a multicenter phase III randomized

controlled clinical study of EORTC/NCIC demonstrated for the first time that temozolomide chemotherapy can significantly prolong the survival time of newly diagnosed GBM patients. At present, the routine 5-day regimen of temozolomide has become the standard postoperative adjuvant chemotherapy for newly diagnosed high-grade glioma patients. Compared with traditional nitrosoureas chemotherapy drugs , temozolomide can be administered orally with fewer adverse reactions, and has been widely used in the treatment of gliomas. In 2009, Groves et al. [20] from the Neuro-Oncology Department of MD Anderson Cancer Center in the United States evaluated temozolomide combined with common IFN -α or polyethylene glycol IFN -α (long-acting IFN -α) in a two-group single-arm clinical phase II study.) for the treatment of adults with relapsed GBM, with an endpoint of 6-month progression-free survival. Adult GBM patients who have relapsed after previous radiotherapy/chemotherapy received standard 5-day regimen of temozolomide (150-200 mg/m^2 , d1-5) combined with IFN -α (4 mIU/m^2 3 times a week) or pegylated IFN -α (0.5 μg / kg , once a week) treatment, and there were 34 cases in the IFN -α group and 29 cases in the polyethylene glycol IFN -α group, the median KPS was 80 points and 90 points, respectively. III/IV The incidence of leukocyte and thrombocytopenia was 35 % and 18 %, respectively, the incidence of grade III/IV fatigue was 18% , and lymphocyte decline was rare. The 6-month progression-free survival rates of the IFN -α group and the pegylated IFN -α group were 31% and 38% , respectively, and the historical comparison showed that it could improve the efficacy of patients with recurrent GBM.

In conclusion, the routine 5-day regimen of temozolomide is currently the standard postoperative adjuvant chemotherapy regimen for newly diagnosed high-grade glioma patients, and IFN -α is the most commonly used interferon for clinical tumor treatment. Our in vitro and in vivo studies have shown that IFN -α can increase the chemosensitivity of TMZ by inhibiting the expression of MGMT and NF-κB in glioma cells, and foreign clinical studies have also shown that IFN -α combined with radiotherapy/chemotherapy for recurrent malignant gliomas has a certain effect. At present, there is no randomized controlled clinical study report on the use of temozolomide combined with IFN -α in the treatment of newly diagnosed high-grade gliomas in home and aboard. The purpose of this study was to observe whether the combined use of interferon on the basis of the current standard temozolomide regimen could further improve the efficacy of newly diagnosed patients with malignant glioma.

Clinical trial design

Research purpose

Main Purpose

Observe and evaluate the effect of temozolomide standard regimen combined with interferon alpha therapy on the overall survival of patients with newly diagnosed high-grade glioma after surgery.

Secondary purpose

Observe and evaluate the effect of temozolomide standard regimen combined with interferon alpha therapy on progression-free survival of patients with newly diagnosed high-grade glioma after surgery.
Observe the safety and tolerability of temozolomide combined with interferon alpha therapy .
Observe the effect of temozolomide combined with interferon alpha therapy on the quality of life of patients with high-grade glioma .

Program Outline

This study is a multicenter, randomized, controlled clinical study of temozolomide combined with interferon alpha in the treatment of newly diagnosed high-grade glioma . The patients will be divided into 2 groups: combined chemotherapy group (temozolomide combined with interferon alpha) and

chemotherapy alone group (temozolomide). To evaluate whether the combined use of interferon alfa based on the current standard temozolomide regimen can further improve the efficacy of newly diagnosed high-grade glioma.

Research centers and cases

The target number of cases in this study was 194, 97 in the combined chemotherapy group and 97 in the chemotherapy alone group .

Research objects

Patients with pathologically confirmed high-grade glioma after surgery .

Inclusion criteria

- o Patients with WHO grade III or IV gliomas confirmed by histopathology after surgery;
- o Patients with no tumor progression after completing concurrent temozolomide chemotherapy/radiotherapy after surgery;
- oAge 18-75 years old;
- o Physical general condition score (KPS) ≥60 ;
- o Expected survival ≥ 2 months;
- o The patient has no major organ dysfunction, and the liver and kidney functions are basically normal. Specific laboratory indicators are required: white blood cells > 4.0×10^{9}/L, platelets > 100×10^{9}/L, hemoglobin > 10 g/dl , Serum bilirubin was lower than 1.5 times the maximum normal value; ALT and AST were lower than 2 times the maximum normal value; serum creatinine was less than or equal to 1.5 mg/dl.

Exclusion criteria

- o Patients who did not receive concurrent temozolomide chemotherapy during postoperative adjuvant radiotherapy;
- o Patients with tumor progression after concurrent temozolomide chemotherapy/radiotherapy;
- o Pregnant and lactating patients;
- o Those with purulent and chronic infectious wounds that do not heal;
- o Liver, kidney and heart insufficiency;
- o Those with a history of uncontrollable mental illness;
- o Those with a history of uncontrolled epilepsy;
- o Those who are considered by the investigator to be inappropriate to participate in this trial.

Rejection criteria

- o Those who violate the entry criteria;
- o Those who did not complete treatment as planned or switched to other drug treatment;
- oThe main indicators are missing and the data are obviously incomplete.

Exit Criteria

- o The patient himself requests to withdraw from the trial;
- o There is a grade 3 or 4 (WHO) adverse reaction that the investigator considers to be related to the combination;
- o Those who cannot be treated according to the plan and have poor compliance;
- o Disease progression.

Test plan

Grouping

This trial is a multicenter, randomized, controlled clinical trial. The ratio of cases in the combined chemotherapy group and the chemotherapy alone group was 1:1. Eligible patients were randomly

divided into the following groups according to the pathological classification (WHO grade III and IV) . The dosing schedule is as follows:

□□ Method of administration

Dosing start time: chemotherapy started within 2-4 weeks after the end of temozolomide concurrent chemotherapy/radiotherapy in both groups.

Combined chemotherapy group:

Temozolomide: 200mg/(m^2.d), orally, d1-5

Interferon alpha: 3mIU/time, subcutaneous injection, d1, d3, d5;

The course of treatment was repeated every 28 days for a total of 12 courses.

Chemotherapy alone group:

Temozolomide: 200mg/(m^2.d), orally, d1-5;

The course of treatment was repeated every 28 days for a total of 12 courses.

□□Observation indicators and inspection time

Checks before enrolling:

- □ Collection of medical history: a complete clinical medical record for the first time, and a brief medical record in the future;
- □ Physical status score (KPS score, see Annex 1), tumor patient quality of life score (QOL) (see Annex 2) and neurological function level evaluation (see Annex 3);
- □ height, weight and vital signs;
- □ Physical examination, especially measurement of palpable or visible lesions;
- □ Blood, urine, stool routine, liver function, renal function and electrolytes, electrocardiogram;
- □ Brain MRI (unenhanced + enhanced + FLAIR) , other examinations such as chest X-ray and B-ultrasound can be selected according to the condition;

Inspections during the study:

During treatment, check and record the following regularly:

Check the content		time
medical history	Including complications and comorbidities	once a week
physical examination	Neurological symptoms should be specifically described	once a week
CBC count	Including hemoglobin, platelets, and white blood cell counts and classifications	once a week
Blood chemistry	Blood Cr, BUN, AST, ALT, total bilirubin, blood electrolytes	Within one week before each treatment
Film degree exam	Brain MRI (unenhanced + enhanced + FLAIR)	Two courses of treatment at least once during the treatment period and once every three months during the follow-up period
ECG	ECG	Within one week before each treatment
other checks	According to clinical needs	According to clinical needs
Adverse reaction assessment	According to WHO standards	once a week

Post-study inspections:

- □ Physical state score (KPS), quality of life score (QOL) of tumor patients , evaluation of nervous system function level;

- weight and vital signs;
- Physical examination, especially measurement of palpable or visible lesions;
- Blood routine, liver and kidney function, blood electrolytes, electrocardiogram;
- Brain MRI (unenhanced scan + enhanced + FLAIR) : At the end of the study and when observing tumor progression in the future, the imaging examination should be the same as the examination method at the time of enrollment, preferably using the same examination equipment.

Follow up

After receiving combined chemotherapy group or the chemotherapy alone group , the toxic and side effects of the patients and the Karnofsky score were evaluated. Follow-up was performed every 2 months until the patient died. We would have follow up by letter or telephone, and make outpatient appointments. The follow-up rate was required to be more than 95%, and the follow-up time was recorded in detail.

Follow-up start and end time

Start after the end of treatment until the patient's death.

Follow-up content

Brain MRI examination, quality of life score (QOL), neurological function level evaluation, etc.

Frequency of follow-up

every 2 months to observe the time to tumor progression (TTP) and overall survival time.

□ Concomitant medication

□□ During the clinical study administration period, other drugs related to tumor treatment should be stopped.

□□ The drugs that can be used concomitantly during the study and their usage are as follows:

- When the adverse reaction caused by the test drug needs to be treated, the symptomatic drug can be given;
- When chemotherapy drugs cause leukopenia and other adverse reactions, related drugs can be given symptomatic treatment;
- when the patient vomits due to chemotherapy;
- Pain medications may be given when the patient's sleep and rest are affected by pain;
- When the patient has constipation, diarrhea, insomnia and other symptoms due to other reasons, symptomatic treatment drugs can be given.

□ Clinical evaluation

The main purpose of this study was to evaluate the effect of the standard regimen of temozolomide combined with interferon alpha therapy on the overall survival and progression-free survival of patients with newly diagnosed high-grade glioma after surgery. To compare the quality of life (QOL), to explore the incidence of adverse reactions, and to further understand the safety and tolerability of temozolomide combined with interferon alpha therapy in clinical applications.

□□ Evaluation method

The main purpose of this study was to evaluate whether the combined use of interferon on the basis of the current standard temozolomide regimen could further improve the efficacy of newly diagnosed high-grade glioma.

□□ Efficacy evaluation

□□□ Observation **of Survival Time**

Survival time refers to the time from the start of chemotherapy to death or the last follow-up time (indicate whether it is still alive).

□□□ Objective curative effect evaluation

If there is residual disease, please refer to Appendix 4: Evaluation Criteria for Neurotumor Efficacy (RANO Criteria)

□□□ Time to **tumor progression** (TTP)

Time to tumor progression (TTP): Refers to the time from when a patient started treatment to the date of any documented disease progression or death from any cause.

□□□ **Karnofsky Score**

Karnofsky physical fitness score of patients before and after treatment (see Annex 1);

□□ □ Quality **of life score of cancer patients (QOL)**

Quality of life score (QOL) of cancer patients (see Annex 2): By comparing the changes in the quality of life scale values before and after treatment, the impact of combined chemotherapy on the quality of life of patients was evaluated.

6.2.6. **Evaluation of the functional level of the nervous system**

Evaluation criteria for the level of nervous system function (see Annex 3): By comparing the changes in the scores of nervous system examinations before and after treatment, the effect of combined chemotherapy on the nervous system function of patients was evaluated.

□□ **Safety evaluation** (see Annex 4)

□□□ **Adverse events**

Definition: Any adverse medical event that occurred between the time a patient was enrolled in the trial and the last follow-up visit, regardless of whether it was causally related to the trial drug, was considered an adverse event.

Adverse events were recorded truthfully during the trial, including the occurrence time, severity, duration, measures taken and outcomes of adverse events.

Adverse event severity judgment criteria: according to the WHO evaluation criteria for adverse drug reactions (Annex 4). If the adverse reactions listed in the table occur, refer to the following expressions:

- ● Mild: does not affect the normal function of the subject.
- ● Moderate: The normal function of the subject is affected to some extent.
- ●Severe: Significantly affects the normal function of the subject.

Judgment criteria for the relationship between adverse events and the trial drug:

The investigator should evaluate the possible association between the adverse event and the investigational drug, with reference to the following criteria:

Certainly related : the time of appearance of the reaction conforms to the chronological order of the medication, the reaction conforms to the known reaction type of the test drug, improves after discontinuation of the drug, and reappears with repeated administration.

Possibly related : the time of occurrence of the reaction corresponds to the chronological order of the medication, the response corresponds to the type of response known to the test drug, and the clinical state of the patient or other treatment modalities may also produce the response.

Possibly irrelevant : The timing of the reaction did not match the chronological order of the medication, the reaction did not match the type of response known to the investigational drug, and the patient's clinical state or other treatment modalities

may have produced the reaction.

Irrelevant: the time of occurrence of the reaction is not in the chronological order of the medication, the reaction is of a known type of reaction that is consistent with the non-trial drug, the clinical state of the patient or other treatment methods may also produce the reaction, the disease state is improved or the reaction is eliminated by stopping other treatment methods, Reaction occurs with repeated use of other treatments.

Unable to determine: the timing of the reaction is not clearly related to the chronological sequence of the medication, the reaction is similar to the known reaction type of the test drug, and other drugs used at the same time may also cause the same reaction.

□□□ Serious adverse events

□□□□ Determination of serious adverse events

- □ die
- □ life-threatening
- □ lead to hospitalization or prolonged hospitalization
- □ permanently or severely disabled
- □ cause birth defects

□□□□ Any serious adverse reaction that occurs during the clinical trial or within 30 days of the last treatment, regardless of whether it is related to the drug or not, should be immediately orally notified within 24 hours to the person in charge of the study, the ethics committee and the sponsor.

□□□□ Investigators should follow up with serious adverse reactions until symptoms disappear or the condition is stable.

□ Statistical methods:

Survival outcomes were calculated using the Kaplan-Meier method. Survival differences were compared using the log-rank test. Univariate and multivariate analyses were conducted using the Cox proportional hazards model to investigate the effects of different survival factors. Chi-square ($\chi 2$) test was used to determine the differences in the incidence of complications and peri-treatment mortality. A two-sided p-value with p values below 0.05 was considered to be statistically significant for all analyses. Statistical analyses were carried out using SPSS 22.0.

□ research plan

Time of case selection: May 2012

Observation end time: July 2016

Research summary time: July 2017

9. Protection of the rights and interests of subjects in clinical trials

Ethics committees and informed consent forms are the main organizations and measures to protect the rights and interests of subjects. Before the start of the clinical trial, the trial protocol needs to be reviewed and approved by the ethics committee of the research unit and signed for approval before it can be implemented. During the clinical trial, any modification of the trial protocol should be approved by the ethics committee before it can be implemented.

Clinical investigators must explain to subjects that participating in clinical trials is voluntary, and at any stage of the trial, they have the right to withdraw from the trial at any time without

discrimination and retaliation, their medical treatment and rights will not be affected, and they can continue to receive other Treatment modalities and treatments. Subjects must be made aware that their participation in the trial and their personal data in the trial are confidential. It is also necessary to inform the subjects of the nature of the clinical trial, the purpose of the trial, the expected possible benefits and the possible risks and inconveniences, other treatment options available to the subjects and the rights of the subjects in accordance with the Declaration of Helsinki and obligations, so that subjects have sufficient time to consider whether they are willing to participate in the trial and sign the informed consent.

10 Clinical study sponsor: China Anti-Cancer Association

11. Clinical trial unit, address, name and qualification of the principal investigator

clinical trial unit	Principal investigator name and qualifications
Department of Neurosurgery/Neuro-oncology, Sun Yat-Sen University Cancer Center	Zhongping Chen MD, PhD
Department of Neurosurgery, The First Affiliated Hospital of Shenzhen University, Shenzhen Second People's Hospital	Taipeng Jiang, MD, PhD
Department of Neurosurgery, Guangdong Sanjiu Brain Hospital, Guangzhou	Hui Ouyang, MD, PhD
Department of Neurosurgery,Shantou Central Hospital, Shantou	Mingfa Liu, MD, PhD
Department of Neurosurgery, Shenzhen People's Hospital, Jinan University	Yongyang Zhao, MD, PhD
Department of Neuro-Oncology, Guangdong Sanjiu Brain Hospital, Guangzhou	Linbo Cai, MD, PhD
Department of Neurosurgery, Tumor Hospital of Harbin Medical University	Jun Su, MD, PhD
Department of Radiotherapy, The Second Hospital of Hebei Medical University, Shijiazhuang	Xiaoying Xue, MD, PhD
Department of Medical Oncology, The First Affiliated Hospital, Jinan University, Guangzhou	Yiming Wang, MD, PhD
Department of Neurosurgery, Tangdu Hospital, Fourth Military Medical University	Gang Li, MD, PhD
Department of Neurosurgery, Huashan Hospital, Shanghai Medical College, Fudan University	Zhiyong Qin, MD, PhD
Department of Neurosurgery,Xinqiao Hospital, Third Military Medical University	Hui Yang, MD, PhD
Department of Oncology, Guangdong Armed Police Corps Hospital Guangzhou	Tao Zhou, MD, PhD
Department of Radiation Oncology，Affiliated Cancer Hospital and Institute of Guangzhou Medical University	Jinquan Liu, MD, PhD
Department of Radiation Oncology，First People's	Xuefeng Hu[19], MD, PhD

Hospital of Fo Shan Affiliated to Sun Yat-sen University, Foshan

12 Accessories

Annex 1: Quality of life scoring criteria (Karnofsky)

Annex 2: Quality of Life Scale Scores

Annex 3: Evaluation Criteria for the Level of Nervous System Function

Annex 4: Evaluation Criteria for Neurotumor Response (RANO Criteria)

Annex 5: WHO Evaluation Criteria for Adverse Reactions

Annex □ Quality of life (Karnofsky) scoring criteria :

Quality of life (Karnofsky) score

(1) Able to carry on normal activity and to work; no special care needed 80 to 100 points are

Normal, no complaints, no evidence of disease. 100 points

Able to carry on normal activities,minor signs or symptoms of disease. 90 points

Normal activities with effort; some signs or symptoms of disease. 80 points

(2) **Unable to work; able to live at home and care for most personal needs; varying amount of assistance needed**

50-70 points of

Cares for self; unable to carry on normal activity or to do active work. 70 points

Requires occasional assistance, but is able to care for most of his personal needs 60 points

Requires considerable assistance and frequent medical care. 50 points

(3) Unable to care for self; requires equivalent of institutional or hospital care; disease may be progressing rapidly.

0 to 40 points of

Disabled; requires special care and assistance. 40 points

Severely disabled; hospital admission is indicated although death not imminent. 30 points

Very sick; hospital admission necessary; active supportive treatment necessary 20 points

Moribund; fatal processes progressing rapidly. 10points

Dead 0 points

Appendix □: Quality of Life Scale Score

QOL) score for cancer patients (1990 version)

1. Appetite: ① Can hardly eat; ② Food intake is less than 1/2 of normal ; ③ Food intake is 1/2 of normal ;
④The food intake is slightly less; ⑤The food intake is normal.

2. Spirit: ①very poor; ②poor ; ③influenced , but sometimes good and sometimes bad; ④good ; ⑤normal , the same as before the illness.

3. Sleep : ① Difficulty falling asleep; ② Very poor sleep; ③ Poor sleep; ④ Slightly poor sleep; ⑤ Generally normal.

4. : ① Often tired **;** ②Feeling weak; ③Sometimes fatigued; ④Sometimes mild fatigue; ⑤No fatigue.

5. Pain : ①Severe pain with passive position; ②Severe pain; ③Moderate pain; ④Mild pain; ⑤No

pain.

6. **Family understanding and cooperation:** ① No understanding at all; ② Poor; ③ Fair; ④ Good understanding and care of the family; ⑤ Good.
7. **Comprehension and cooperation of colleagues (including leaders):** ① No understanding at all, no one takes care of them; ② Poor; ③ Average; ④ Few people understand and care ;
8. **Self-understanding of cancer** : ① Disappointed, completely uncooperative; ② Uneasy, reluctant to cooperate; ③ Uneasy, generally cooperative; ④ Uneasy, but able to cooperate well; ⑤ Optimistic, confident.
9. **Attitudes towards treatment** : ① No hope for treatment; ② Doubt about treatment; ③ Hope to see curative effect, but fear of side effects; ④ Hope to see curative effect and still cooperate;
10. **Daily life** : ① bedridden; ② able to move, most of the time need to stay in bed; ③ able to move, sometimes bed rest; ④ normal activities, unable to work; ⑤ normal activities and work.
11. **Side effects of treatment** : ① Seriously affect daily life; ② Influence daily life; ③ After symptomatic treatment, it can not affect daily life; ④ No symptomatic treatment basically does not affect daily life; ⑤ Does not affect daily life.
12. **Facial expressions (** as shown) Facial expressions are scored as follows:

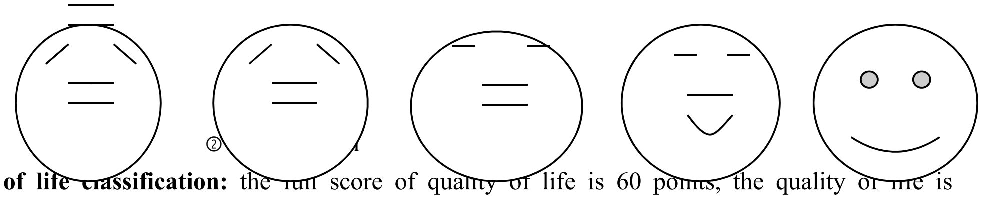

② [illegible]

Quality of life classification: the full score of quality of life is 60 points, the quality of life is extremely poor, it is <20 , the poor is 21-30 , the general is 31-40 , the better is 41-50 , and the good is 51-60 point.

(Refer to the relevant chapters in "Guiding Principles of Cancer Three-step Pain Relief", 1st edition, August 1999, edited by Sun Yan)

Annex 3. Evaluation criteria for the level of nervous system function

<table>
<tr><td rowspan="4">nervous system an examination</td><td>Spirit state</td><td>Response: □ normal □ slow; timing orientation: □ accurate □ wrong;
Memory: □ normal □ poor □ very poor</td></tr>
<tr><td>language</td><td>□ Normal; Aphasia: (□ Motor □ Sensory □ Named □ Mixed)</td></tr>
<tr><td>sports</td><td>□ Normal; Paralyzed: (□ Left upper limb □ Left lower limb □ Right upper limb □ Right lower limb)</td></tr>
<tr><td>Paralyzed limb muscle strength</td><td>□ Class 0 □ Class I □ Class II □ Class III □ Class IV □ Class V</td></tr>
<tr><td>nervous system check score</td><td colspan="2">functional level</td></tr>
<tr><td>□ 0</td><td colspan="2">No neurological impairment, fully autonomous at home or work without assistance</td></tr>
<tr><td>□ 1</td><td colspan="2">Mild neurological deficit, complete autonomy at home or work without assistance</td></tr>
<tr><td>□ 2</td><td colspan="2">Moderate neurological impairment, fully autonomous at home or work, but needs help</td></tr>
<tr><td>□ 3</td><td colspan="2">Moderate neurological impairment, lack of full autonomy at home or work, and needing help</td></tr>
<tr><td>□ 4</td><td colspan="2">Severe neurological impairment, complete inactivity at home or treatment center, needing help; inability to work</td></tr>
<tr><td>□ 9</td><td colspan="2">unknown</td></tr>
</table>

Annex 4. Evaluation Criteria for Neurotumor Efficacy (Response Assessment in Neuro-Oncology Working Group , RANO)

RANO standard

1. Complete remission (CR):complete disappearance of all enhancing measurable and nonmeasurable disease sustained for at least 4 weeks;no new lesions; stable or improved nonenhancing(T2/FLAIR) lesions; patients must be off corticosteroids;and and stable or improved clinically

2. Partial remission (PR): ≥50% decrease compared with baseline in the sum of products of perpendicular diameters of all measurable enhancing lesions sustained for at least 4 weeks; no progression of nonmeasurable disease; no lesions; stable or improved nonenhancing (T2/FLAIR)on same or lower dose of corticosteroid dose at the time of the scan evaluation should be no greater than the dose at time of baseline scan; stable or improved clinically

3. Stable disease (SD): The product of the two diameters of the tumor-enhancing lesions on T1 is reduced by ≥25%, but less than 50%, no new lesions appear, and maintained for more than 4 weeks; the range of non-enhancing lesions on T2/FLAIR is stable or reduced ; Stable or reduced hormone dose; Stable or improved clinically

4. Progression disease (PD): ≥25% increase in sum of the products of perpendicular diameters of enhancing lesions or on increasing doses of corticosteroids; a significant increase in T2/FLAIR nonenhancing lesions;the appearance of any new lesions; or definite clinical deterioration not attributable to other causes apart from tumor

Annex 5. WHO evaluation criteria for adverse events

WHO evaluation criteria for adverse events

Toxic and side effects indicators	Grading				
	0	I	II	III	IV
**Blood System (Adult)**					
White blood cells (x10 ^{3} /L)	≥4.0	3.0-3.9	2.0-2.9	0-1.9	<1.0
Platelets (x10 ^{3} /L)	≥100	75-99	50-74	36-40	<36
Granulocytes (x10 ^{3} /L)	≥2.0	1.5-1.9	1.0-1.4	0.5-0.9	<0.5
Hemoglobin (g/L)	≥110	95-109	80-94	65-79	<65
bleeding	none	ecchymosis	mild blood loss	moderate blood loss	Severe blood loss is life-threatening

**digestive system**					
Nausea, vomiting	none	nausea	vomiting, manageable	Frequent vomiting requiring treatment	Frequent vomiting that is difficult to control
Diarrhea	none	ephemeral (2d)	Tolerable (2d)	intolerable, need treatment	Dehydration or bloody diarrhea
Bilirubin (kmol/L)	≤1.25XN	1.26 - 2.5XN	2.6 - 5XN	5.1-10XN	>10XN
Transaminase (kmol/L)	≤1.25XN	1.26 - 2.5XN	2.6 - 5XN	5.1-10XN	>10XN
Stomatitis	none	pain, erythema	Ulcers can eat	ulcer, fluid	Ulcer, can't eat
**urinary system**					
Creatinine (kmol/L)	≤1.25XN or ≤106.08	1.25-2.5XN or 114.92-176.8	2.6-5XN or 185.64-353.6	5—10XN or >353.6	>10XN or symptomatic uremia
Urea ammonia (kmol/L)	≤1.25XN or ≤7.14	1.25—2.5XN or 7.5-14.28	2.6-5XN or 14.64-21.42	5—10XN or >21.42	>10XN or symptomatic uremia
proteinuria	none	+,<3g/L	++3—10g/L	+++>10g/L	++++, Nephrotic Syndrome
hematuria	none	microscopic hematuria	gross hematuria	Gross hematuria with blood clots	Concurrent urinary tract obstruction
**Peripheral nervous system**	none	Paresthesia and/or decreased tendon reflexes	Severe paresthesia and/or weakness	Intolerance affected by abnormal or marked dyskinesia	paralysis
constipation	none	mild	Moderate	bloating	Bloating with vomiting (paralytic ileus)
lung	none	mild symptoms	Difficulty breathing after activity	Difficulty breathing at rest	Absolute bed rest
**heart**					
heart arrhythmia	none	Sudden, heart rate > 110 beats/min	Unifocal premature beat (atrial)	multifocal premature beat	severe heart rhythm disorder
Cardiac Function	normal	Asymptomatic, with abnormal signs	Transient cardiac insufficiency without treatment	Symptomatic cardiac function, insufficiency , requiring treatment	congestive heart failure
skin	no change	Erythema, hyperpigmentati on	Dry peeling blisters, itching	wet peeling ulcer	exfoliative inflammation with necrosis
hair loss	none	mild	Moderate	Fully detachable,	Completely detached,

				renewable	non-regenerating
drug fever	none	<38 ℃, <4 h	38 ℃ -40 ℃ ,>4h	>40 ℃ , >4h	Fever with drop in blood pressure
Infection	none	mild	Moderate	severe	Life-threatening with drop in blood pressure
Fatigue	none	Fatigue, does not affect normal life	Sometimes bedridden, does not affect normal life	Most of the time in bed, affecting normal life	bedridden, affecting normal life
allergy	none	edema	Bronchospasm without treatment	Bronchospasm, requiring treatment	anaphylactic shock
pain	none	mild	Moderate	severe	intractable pain

Note: N is the normal value

references

1. Chinese Medical Association Neurosurgery Branch Tumor Professional Group. Chinese consensus on the diagnosis and treatment of malignant gliomas in the central nervous system (simplified version). Chinese Medical Journal, 2009, 89(43) .
2. Stupp R, Mason W P, van den Bent M J, et al. Radiotherapy plus concomitant and adjuvant temozolomide for glioblastoma. N Engl J Med , 2005 , 352: 987 -996.
3. Stupp R , Hegi ME , Mason WP , et al. Effects of radiotherapy with concomitant and adjuvant temozolomide versus radiotherapy alone on survival in glioblastoma in a randomised phase III study: 5-year analysis of the EORTC-NCIC trial. Lancet Oncol, 2009, 10(5): 459-466.
4. Compiled by the Neuro-Oncology Professional Committee of the Chinese Anti-Cancer Association: Outline of Diagnosis and Treatment of Common Tumors of the Central Nervous System. Published by Peking University Medical Press, May 2010.
5. Hegi Me, Liu L, Herman JG, et al. Correlation of O6-methylguanine methyltransferase (MGMT) promoter methylation with clinical outcomes in glioblastoma and clinical strategies to modulate MGMT activity. J Clin Oncol , 2008 , 26 : 4189–4199.
6. Xu Hongsheng, Wang Zhimin, Chen Zhongping. Research progress of MGMT expression and chemoresistance in brain tumors. Chinese Journal of Neuro-Oncology, 2005, 3: 30-34.
7. Lavon I, Fuchs D, Zrihan D, et al. Novel mechanism whereby nuclear factor kappaB mediates DNA damage repair through regulation of O(6)-methylguanine-DNA-methyltransferase. Cancer Res, 2007, 67 (18): 8952-8959.
8. Maher SG , Romero-Weaver AL , Scarzello AJ , et al. Interferon: cellular executioner or white

knight? Curr Med Chem , 2007 , 14(12): 1279-1289 .

9. Olson JJ , James CD , Lawson D , et al. Correlation of the response of recurrent malignant gliomas treated with interferon alpha with tumor interferon alpha gene content. Int J Oncol , 2004 , 25(2):419-4 27 .
10. Sondak VK. How does interferon work ? Does it even matter? Cancer, 2002, 95(5): 947-949.
11. Shen Dong, Qiu Zhikun, Chen Yinsheng, et al. Interferon-α/β sensitizing effect of temozolomide on MGMT-positive glioma stem cells in vitro. Journal of Sun Yat-Sen University Medical Edition, March 2012
12. Wang Jie, Shen Dong, Chen Furong, et al. Experimental study of temozolomide combined with interferon α/β in the treatment of glioma xenografts. Chinese Journal of Neuro-Oncology, 2011, 9(3): 147-151.
13. Liao Meilin, Zhou Yunzhong, Zhao Sen, et al. Research on the optimization of comprehensive treatment of lung cancer and molecular biology. China Oncology, 1996, 5(7): 3-4.
14. Yang Xuening, Wu Yilong, Wang Siyu. et al. A randomized controlled clinical study of interferon-α therapy after complete resection of stage I and II non-small cell lung cancer. China Journal of Lung Cancer, 2003, 6(5): 339-342.
15. Dillman RO , Wiemann M , Oldham RK , et al. Interferon alpha-2a and external beam radiotherapy in the initial management of patients with glioma: a pilot study of the National Biotherapy Study Group. Cancer Biother, 1995, 10(4): 265 -271.
16. Buckner JC, Brown LD, Kugler JW, et al. Phase II evaluation of recombinant interferon alpha and BCNU in recurrent glioma. J Neurosurg, 1995, 82: 430-435.
17. Rajkumar SV , Buckner JC , Schomberg PJ , et al. Phase I evaluation of radiation combined with recombinant interferon alpha-2a and BCNU for patients with high-grade glioma. Int J Radiat Oncol Biol Phys, 1998, 40(2): 297- 302.
18. Brandes AA , Scelzi E , Zampieri P , et al. Phase II trial with BCNU plus alpha-interferon in patients with recurrent high-grade gliomas. Am J Clin Oncol. 1997, 20(4):364-367.
19. Buckner JC , Schomberg PJ , McGinnis WL , et al.A phase III study of radiation therapy plus carmustine with or without recombinant interferon-alpha in the treatment of patients with newly diagnosed high-grade glioma. Cancer, 2001, 92(2): 420-433.
20. Groves MD, Puduvalli VK, Gilbert MR, et al.Two phase II trials of temozolomide with interferon-alpha2b (pegylated and non-pegylated) in patients with recurrent glioblastoma multiforme. Br J Cancer, 2009, 101(4):615-620 .
21. Patrick Y. Wen, David R. Macdonald, et al. Updated Response Assessment Criteria for High-Grade Gliomas: Response Assessment in Neuro- Oncology Working Group . J Clin Oncol , 2010 , 28(11):1963-1972 .
