# Supplementary material for: Adjuvant Temozolomide Chemotherapy With or Without Interferon Alfa Among Patients With Newly Diagnosed High-grade Gliomas: A Randomized Clinical Trial
Source: JAMA Netw Open. 2023 Jan 27;6(1):e2253285. doi: 10.1001/jamanetworkopen.2022.53285 (PMC11839150; doi:10.1001/jamanetworkopen.2022.53285)
Supplement: Supplement 2. — eTable 1. Participating Institution and Researchers eTable 2. Toxic Effects of the Patients in Temozolomide Plus Interferon Alfa Chemotherapy Cohort and Temozolomide Cohort eTable 3. Bivariable and Multivariable Analysis for OS and PFS eMethods. The Detection for the Methylation Status of the MGMT Promoter eFigure. Molecular Signatures Related to Response to Temozolomide With Interferon Alfa Therapy [file jamanetwopen-e2253285-s002.pdf]

Supplementary Online Content

Guo C, Yang Q, Xu P, et al. Adjuvant temozolomide chemotherapy with or without interferon alfa among patients with newly diagnosed high-grade gliomas: a randomized clinical trial. *JAMA Netw Open*. 2023;6(1):e2253285. doi:10.1001/jamanetworkopen.2022.53285

eTable 1. Participating Institution and Researchers

eTable 2. Toxic Effects of the Patients in Temozolomide Plus Interferon Alfa Chemotherapy Cohort and Temozolomide Cohort

eTable 3. Bivariable and Multivariable Analysis for OS and PFS

eMethods. The Detection for the Methylation Status of the MGMT Promoter

eFigure. Molecular Signatures Related to Response to Temozolomide With Interferon Alfa Therapy

This supplementary material has been provided by the authors to give readers additional information about their work.

© 2023 Guo C et al. *JAMA Network Open*.

eTable 1. Participating Institution and Researchers

	Institution	Investigator
	Sun Yat-sen University Cancer Center	Zhongping Chen<br>Qunying Yang<br>Chengcheng Guo
	Shenzhen Second People's Hospital	Taipeng Jiang
	Southern Hospital of Southern Medical University	Hui Ouyang
	Shantou Central Hospital	Mingfa Liu
	Shenzhen Second People's Hospital	Yongyang Zhao
	Guangdong Sanjiu Brain Hospital	Linbo Cai
	Affiliated Tumor Hospital of Harbin Medical University	Jun Su
	The second hospital of Hebei Medical University	Xiaoying Jiang
	The First Affiliated Hospital of Jinan University	Yiming Wang
	Tangdu Hospital, the fourth military medical university	Gang Li
	Huashan Hospital, Fudan Univiersity	Zhiyong Qin
	Xinqiao Hospital, the third Military Medical University	Hui Yang
	Guangdong Armed police Corps Hospital	Tao Zhou
	Cancer Hospital Affiliated to Guangzhou Medical University	Jinquan Liu
	Foshan first people's Hospital	Xuefeng Hu

© 2023 Guo C et al. *JAMA Network Open*.

eTable 2. Toxic Effects of the Patients in Temozolomide Plus Interferon Alfa Chemotherapy Cohort and Temozolomide Cohort

	TMZ+IFN（n=100）	TMZ（n=99）	P 值
	(%)	例(%)	
Anemia			
I-II	8（8.0）	9（9.1）	0.78
III	3（3.0）	2（2.0）	0.66
Leukopenia			
I-II	16（16.0）	15（15.2）	0.87
III	5（5.0）	5（5.1）	0.99
thrombocytopenia			
Ⅰ-Ⅱ	4（4.0）	5（5.1）	0.72
Liver Dysfunction			
I-II	6（6.0）	9（9.1）	0.41
III	1（1.0）	1（1.0）	0.99
Nausea / vomiting			
Ⅰ-Ⅱ	6（6.0）	7（7.1）	0.76
constipation			
Ⅰ-Ⅱ	25（25.0）	36（36.4）	0.08
fatigue			
I-II	8（8.0）	4（4.0）	0.24
III	1（1.0）	0（0.0）	0.32
Fever			
I-II	5（5.0）	0（0.0）	0.02
III	1（1.0）	0（0.0）	0.32
Dermal toxicity			
Ⅰ-Ⅱ	1（1.0）	2（2.0）	0.56
epilepsy	2（2.0）	0（0.0）	0.16

eTable 3. Bivariable and Multivariable Analysis for OS and PFS

**OS**	**HR (bivariable)**	**HR (multivariable)**
**Age**	1.01 (0.99-1.02, p=0.314)	1.00 (0.98-1.01, p=0.763)
**Gender**		
	1.01 (0.74-1.37, p=0.970)	0.92 (0.67-1.27, p=0.606)

© 2023 Guo C et al. *JAMA Network Open*.

**KPS**		
	0.99 (0.97-1.00, p=0.069)	0.98 (0.97-1.00, p=0.024)
**MGMT**		-
	0.74 (0.54-1.00, p=0.050)	0.61 (0.44-0.84, p=0.003)
**Grade**		-
	2.63 (1.91-3.63, p<0.001)	2.99 (2.09-4.27, p<0.001)
**Total resection**		-
	1.87 (1.37-2.55, p<0.001)	1.97 (1.38-2.81, p<0.001)
**Cycle>6**	-	-
	0.54 (0.39-0.74, p<0.001)	0.84 (0.58-1.22, p=0.365)
**Group**	-	-
	0.64 (0.47-0.88, p=0.005)	0.65 (0.47-0.89, p=0.008)

**PFS**	**HR (Bivariable)**	**HR (Multivariable)**
**Age**	1.00 (0.99-1.01, p=0.839)	0.99 (0.98-1.00, p=0.205)
**Gender**	-	-
	0.96 (0.71-1.29, p=0.768)	0.84 (0.62-1.15, p=0.287)
**KPS**	0.99 (0.98-1.01, p=0.424)	0.99 (0.97-1.00, p=0.124)
**MGMT**	-	-
	0.84 (0.63-1.13, p=0.253)	0.85 (0.62-1.14, p=0.276)
**Grade**	-	-
	2.67 (1.96-3.65, p<0.001)	2.46 (1.76-3.44, p<0.001)
**Total resection**	-	-
	1.75 (1.29-2.36, p<0.001)	1.50 (1.06-2.11, p=0.020)
**Cycle>6**	-	-
	0.40 (0.29-0.54, p<0.001)	0.55 (0.38-0.78, p=0.001)
**Group**	-	-
	0.79 (0.59-1.06, p=0.116)	0.90 (0.67-1.23, p=0.521)

eMethods. The Detection for the Methylation Status of the MGMT Promoter

The methylation status of the MGMT promoter was also analysed using methylation-specific polymerase chain reaction (MS-PCR), and the results were compared with those of methylation-specific multiplex ligation-dependent probe amplification (MS-MLPA). The prepared DNA was modified by sodium bisulfite treatment using an EZ DNA Methylation-Gold Kit (Catalogue No. D5005; Zymo Research, Orange, CA). The

© 2023 Guo C et al. *JAMA Network Open*.

primer sequences used for MGMT were as follows: methylated forward, 5′ TTT CGA CGT TCG TAG GTT TTC GC 3’; methylated reverse, 5’ GCA CTC TTC CGA AAA CGA AAC G 3’; unmethylated forward, 5’ TTT GTG TTT TGA TGT TTG TAG GTT TTT GT 3’; and unmethylated reverse, 5′ AAC TCC ACA CTC TTC CAA AAA CAA AAC A 3’. The obtained polymerase chain reaction products were electrophoresed on 2% agarose gels and visualized under UV illumination after staining with ethidium bromide. The assay results were evaluated, as described previously[17].

Whole Exome Sequencing

To identify molecular features that were significantly enriched in either responsive or non-responsive tumors, we collected tumor samples and bloods from 20 patients of TMZ+IFN group, which divided into responder group and non-responder group. Patients were classified as responders if tumor were either stable or shrinking continually over at least 6 courses of treatment. The tumor tissues and matching blood samples analyzed in this study were obtained from the biospecimen bank of SYSUCC. All tissues and bloods were collected with written informed patient consent. Sequencing libraries were generated using Agilent SureSelect All Exon V5 kits and sequenced on the HiSeq2500 platform to generate 150 bp reads. After quality control, paired reads were mapped to the human reference genome hg19 using BWA-MEM (v.0.7.12) with default parameters[18]. Samtools[19], Picard, and GATK were used to sort BAM files and do duplicate marking, local realignment, and base quality recalibration to generate final BAM files. Somatic SNVs were identified using MuTect (v.1.1.7)[20] for each tumor/blood pair. Somatic indeles were called using Strelka (v.1.0.15)[21] with default parameters. Filtered sSNVs and indels were annotated with ANNOVAR[22].

© 2023 Guo C et al. *JAMA Network Open*.

Finaly, Sequenza (v.2.1.2)[23] was used to estimate both the copy number profile and tumor purity, and ploidy for each sample. Default settings were used following the recommendations of the manual.

DNA Methylation Analysis

Illumina Methylation Beadarray 850K flatform data were processed using the ChAMP (v.2.26.0)[24] package. In brief, probes were filtered based on default parameters. Methylation at each locus was reported as a beta value, ranging from 0 to 1. Beta mixture quantile (BMIQ) normalization was applied for normalization of the data. Differentially methylated positions (DMPs) between each group were detected. We identified DMPs at the significance of Benjamini–Hochberg correction with adjusted $P < 0{\cdot}05$.

RNA-seq Data Analysis

A total of 13 tumor tissues were performed RNA sequencing. The sequencing reads were aligned to the human reference sequence (hg38 assembly) using Salmon[25]. Differential expression analysis was performed using DESeq2[26] (v.1.36.0). Gene expression values were quantitated as transcripts per million reads (TPM) values for visualization. Gene set enrichment analysis (GSEA) was performed to identify phenotype-related biological processes.

© 2023 Guo C et al. *JAMA Network Open*.

eFigure. Molecular Signatures Related to Response to Temozolomide With Interferon Alfa Therapy

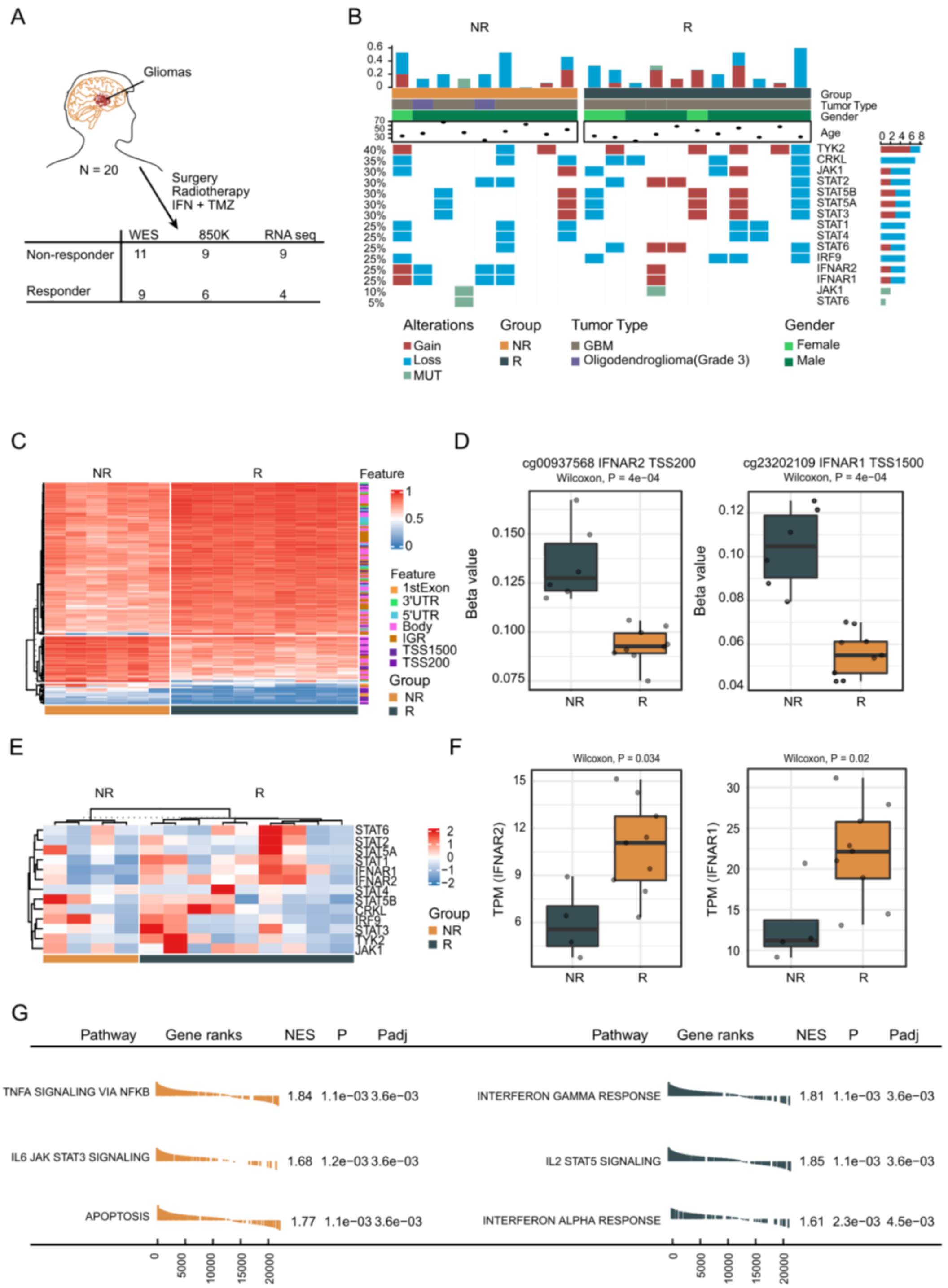

© 2023 Guo C et al. *JAMA Network Open*.
