# Supplementary material for: Adjuvant Temozolomide Chemotherapy With or Without Interferon Alfa Among Patients With Newly Diagnosed High-grade Gliomas: A Randomized Clinical Trial
Source: JAMA Netw Open. 2023 Jan 27;6(1):e2253285. doi: 10.1001/jamanetworkopen.2022.53285 (PMC11839150; doi:10.1001/jamanetworkopen.2022.53285)
Supplement: Supplement 3. — Data Sharing Statement [file jamanetwopen-e2253285-s003.pdf]

Data Sharing Statement

Guo. Adjuvant Temozolomide Chemotherapy With or Without Interferon Alfa Among Patients With Newly Diagnosed High-grade Gliomas. *JAMA Netw Open*. Published January 27, 2023. doi:10.1001/jamanetworkopen.2022.53285

Data
Data available: No
